# Activated Carbon from Banana Pseudostem: Multivariate Optimization of Synthesis and Adsorption Study for Phosphorus Removal

**DOI:** 10.1021/acsomega.5c08820

**Published:** 2026-01-01

**Authors:** Moisés de Souza Luz Faria, Tatianny de Araujo Andrade, Renata Pereira Lopes Moreira, Rita de Cássia Superbi de Sousa, Alisson Carraro Borges

**Affiliations:** † Department of Chemistry, 28120 Federal University of Viçosa, Ave. P.H Rolfs, s/n, Viçosa, MG 36570-900, Brazil; ‡ Department of Agricultural Engineering, Federal University of Viçosa, Ave. P.H Rolfs, s/n, Viçosa, MG 36570-900, Brazil

## Abstract

This work aimed to develop a high-surface-area activated carbon using banana pseudostem as precursor and zinc chloride as a chemical activator, with synthesis conditions optimized via a central composite rotational design (CCRD) for phosphate adsorption. The resulting banana pseudostem activated carbon (BPAC) exhibited an exceptionally high specific surface area of 2415 m^2^ g^–1^, obtained at activation temperature (AT) of 400 °C, 2:1 impregnation ratio (IR), and pyrolysis time (PT) of 1 h in an air atmosphere muffle furnace. This result exceeds the CCRD-predicted value (1037 m^2^ g^–1^), highlighting the importance of post-pyrolysis factors, such as the duration of contact with the HCl soaking solution employed during the biochar cleaning stage. The pH at the point of zero charge (7.30) suggests that phosphate adsorption is favored below this value. X-ray diffraction analysis confirmed a primarily amorphous structure with a remaining lamellar zinc monohydrate peak. The CCRD demonstrated a significant effect of PT, IR, and AT. Batch adsorption assays showed that phosphate adsorption followed the pseudo-second-order kinetics, reaching equilibrium in approximately 7 h, and the Langmuir model fitted best, with a maximum adsorption capacity of 11.78 mg g^–1^.

## Graphical abstract

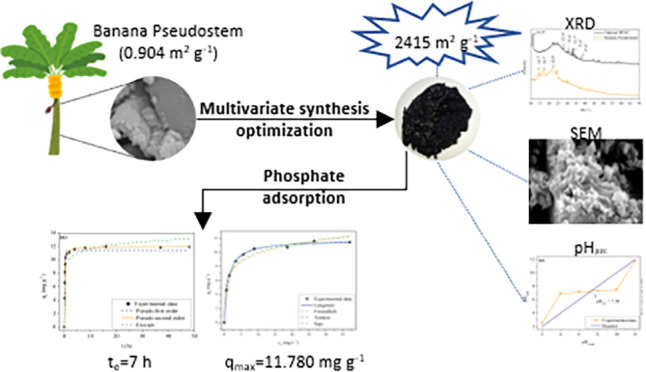

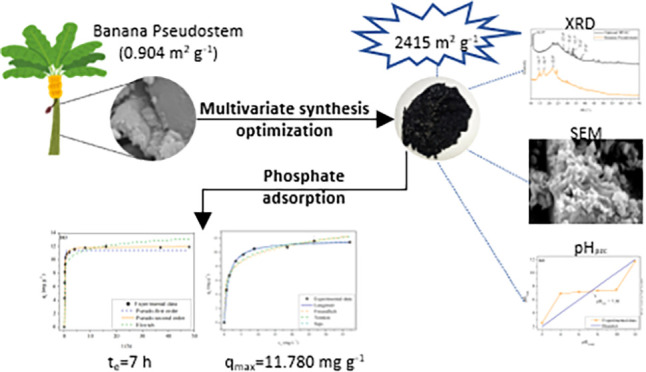

## 1 Introduction

Irregular disposal of wastewater causes negative impacts on the environment, serious damage to human health, and aquatic pollution. Irresponsible human activities in rivers and lakes contribute to their contamination and degradation. Furthermore, the growth of the world population tends to increase the demand for clean water.^1^ Fertilization activities, animal residues, and inefficient domestic wastewater treatment enhance the eutrophication levels in water bodies due to their nitrogen and phosphorus content.^2,3^ This environmental problem is commonly associated with fast harmful algae growth, which stimulates the surface covering in water systems, leading to oxygen depletion and light obstruction.^3^ A variety of alternatives are commonly employed for the treatment of wastewater, such as sedimentation, coagulation, biological techniques, reverse osmosis, and ionic exchange. However, some of these methods may be expensive or cause secondary pollution.^4^ Adsorption has been studied in the last decades as a promising technique for the removal of many contaminants, including phosphorus, with different materials being employed as adsorbents.^4–7^ This technique promotes advantages compared to other treatment methods, for example, low cost, high effectiveness, easy operation, and fewer byproducts.^8^

Various materials have been developed to enhance the adsorption of contaminants in aqueous solutions. The synthesis of iron-based nanocomposites on activated carbon has shown versatile applications in the removal of toxic metals and dyes, due to the combination of high surface area and active functional groups, acting through electrostatic interactions between the adsorbent and the adsorbate.^9^ The use of lignocellulosic residues, such as pea pods and watermelon peels, has also been explored as a sustainable approach for producing chemically activated porous matrices, which are effective at removing Cu^2+^ ions. This performance is enhanced by the incorporation of functional groups and is influenced by factors such as adsorbent dosage, pH, contact time, and the initial concentration of the contaminant.^10,11^

Similar research efforts have focused on creating functional materials, such as three-dimensional electrocatalytic systems based on metal organic frameworks (MOFs), for an efficient degradation of pollutants like norfloxacin.^12^ The development of novel nanocomposites has also attracted attention, particularly those combining activated carbon and nanocellulose as the shell with a core formed by cationic metal oxide nanoparticles, enabling the efficient removal of bicarbonate ions.^13^

Phosphorus is an essential nutrient for the agricultural industry. However, its natural reserves are nonrenewable, which highlights the need to develop effective materials capable of capturing its ionic forms from wastewater, since the release of phosphorus into water bodies represents a major environmental concern.^14^ Therefore, the development of effective adsorbents, such as activated carbon, is crucial to a sustainable approach. Furthermore, the use of alginate hydrogels has been reported as another efficient adsorbent, since the modification of surface −OH and −COOH groups promotes an enhancement in phosphate removal.^15^ Significant progress has also been achieved in biomass-derived sorbents for phosphate remediation as observed in the modification of rice husk biochar with MgAl_2_O_4_.^16^

In this context, adsorption stands out as an advantageous technique for phosphorus removal due to its efficiency and the possibility of regenerating adsorbents.^17^ Phosphate ion removal by adsorption is influenced by the pH value, which determines the electrostatic interactions, by selectivity, since different ions compete for the active sites, and by the initial concentration of phosphorus in the form of phosphate.^18^

Lignocellulosic residues are carbon-rich resources that can be recycled for the synthesis of functional materials. Controlled slow pyrolysis is a process based on the thermal degradation of the source material, resulting in a highly porous product, named biochar.^19^ Biochar may enhance the phosphorus adsorption capacity compared to other adsorbents due to its high specific surface area (*S*_BET_), porous structure, and stable chemical properties.^18^ The specific surface area of biochars can be improved using different activators, such as physical or chemical reactants.^20^ Zinc chloride (ZnCl_2_) is a chemical reactant widely used for activation of carbonaceous material for developing high specific surface area structures and porosity formation compared to sodium hydroxide, sulfuric acid, and phosphoric acid.^21–23^ Acting as a Lewis acid, this salt induces dehydration of lignocellulosic residues, thereby enhancing the decomposition of their structure during pyrolysis.^24^ Innumerable lignocellulosic residues have been studied for activated carbon production, such as sugar cane bagasse,^25^ coffee waste,^26^ banana peel,^27^ cassava starch waste,^28^ baobab husk,^29^ and banana pseudostem (BP).^7,30,31^

Each ton of banana fruit generates four tons of residues, from which the majority is composed of BP (*Musa* spp.). This residue may be burned after fruit harvesting or even discharged without proper treatment, promoting the eutrophication of water bodies and negatively impacting the environment.^32^ Furthermore, the discharge of BP means the waste of potential and valuable resources.^33^ BP is a natural fibrous material with great potential as a precursor of high-quality carbon-rich structures, whose properties may be influenced by different parameters such as activation temperature, pyrolysis time, and activation methods, which affect, for example, the development of surface area.^34^ The morphological and structural properties of BP make it a promising precursor for developing porous carbon materials with a high surface area and tunable active sites. Similar approaches have been applied in catalytic degradation systems, where the structural tailoring of nanocomposites such as Sb_2_O_3_–CuO has been shown to enhance pollutant degradation efficiency.^35^

Although conventional methods for producing activated carbons may yield efficient adsorbents, a systematic study of the production criteria can generate materials with an even higher efficiency by optimizing various factors. On a large scale, this translates into economic and sustainable benefits, as improved process parameters can reduce costs while maintaining an efficient product.^36^ The Central Composite Rotational Design (CCRD) is a methodology within Response Surface Methodology (RSM) and represents a simultaneous approach for optimizing multiple factors affecting a dependent variable, making it ideal for evaluating and enhancing adsorption systems.^37^ Variables such as activation temperature (AT), pyrolysis time (PT), and impregnation ratio (IR) have been applied in RSM for the synthesis of activated carbon derived from hazelnut shells to evaluate their effects on yield and specific surface area,^36^ while the production of hydrogen was also investigated using this methodology, varying the temperature, methane concentration, and catalyst quantity.^38^ At the laboratory bench, this also allows a reduction in the number of experiments and enables a multifactor statistical analysis to determine the ideal conditions for all relevant parameters.^39,40^ However, many studies still lack a methodology that enables simultaneous optimization of parameters for the synthesis of biochars. In the case of BP, this potential remains underexplored, particularly regarding synthesis under an oxygen-containing atmosphere, selection of an optimal point based on minimal energy consumption, and aiming the material for phosphate adsorption.

Therefore, this study aimed to optimize the production of banana pseudostem activated carbon (BPAC) using ZnCl_2_ as a chemical activator and an air atmosphere muffle furnace for phosphorus adsorption. The synthesis of BPAC was optimized, for the first time, using a CCRD methodology within the Response Surface Methodology framework, combining the effects of the activation temperature, ZnCl_2_ impregnation ratio, and pyrolysis time on the specific surface area. The optimal conditions were based on the lower levels of the independent factors, maximizing specific surface area values, and a simplified cost-benefit analysis based on the costs of reactant and energy compared to the maximum surface area BPAC obtained by RSM. The optimal BPAC was characterized using techniques such as specific surface area analysis, pore size distribution, X-ray diffraction, scanning electron microscopy, pH of point of zero charge, and ζ-potential. Finally, the kinetic and isotherm batch studies were conducted on the optimal BPAC at 20 °C to determine its equilibrium time and adsorption capacity of phosphate.

## 2 Materials and Methods

### 2.1 Material

The assays in this study used analytical-grade chemical reagents as follows: hydrochloric acid (HCl P.A. Fmaia, purity 37%); sodium hydroxide microspheres (NaOH P.A. Vetec Química, purity 98%); ascorbic acid (C_6_H_8_O_6_ P.A. RM Maia, purity 99%); ammonium molybdate (N_6_H_24_Mo_7_N_6_O_24_·4H_2_O P.A., Êxodo Científica, purity 98%); potassium antimony tartrate (K_2_(C_4_H_2_O_6_Sb)_2_·3H_2_O P.A., ACS Científica, purity 99%); monopotassium phosphate (KH_2_PO_4_ P.A. Dinâmica, purity 99%); and anhydrous zinc chloride (ZnCl_2_ P.A. Êxodo Científica, 96% purity).

### 2.2 Banana Pseudostem Activated Carbon Synthesis

BPAC was developed following the methodology of Faria et al. (2025).^7^ The banana pseudostem (*Musa* spp.) was collected in the Experimental Area of Waste Treatment in the Department of Agricultural Engineering at the Federal University of Viçosa, Minas Gerais, Brazil. The residue was collected after fruit harvesting. The lignocellulosic biomass was cut into 3–5 cm small pieces, and then dried in an air-forced oven (Marconi, MA035) at 65 °C for 7 days for residual humidity removal.

The dried banana pseudostem was milled using a processor (Philco, PH900 Turbo, 250 W, single speed). Approximately 500 mL of dried BP small pieces were placed in the container and milled for 2 min. After milling, the material was sieved through a 35-mesh sieve. The retained fraction was milled again, while the portion passing through the 35-mesh sieve was impregnated with ZnCl_2_ solution. The impregnation consisted of hand shaking using a glass rod to guarantee the homogeneous aspect of BP and ZnCl_2_ solution. The IR used in this study varied according to eq (S1) in the Supporting Information (SI). The IR is defined as the mass of anhydrous ZnCl_2_ divided by the mass of banana Pseudostem and is a dimensionless quantity. For each crucible, a fixed 15 g mass of BP was used. The delimitated amount of ZnCl_2_ was diluted in 50 mL of deionized water before being mixed with the precursor. After that, the resulting mixture was dried at 85 °C for 24 h using an oven (Orion, 515).

After this step, the impregnated BP was allowed to reach room temperature, and the crucible was capped with a porcelain lid and then placed in a muffle furnace (SP Labor, SP-1200-3.5 kW) for pyrolysis and development of BPAC in an air atmosphere. Under a 10 °C min^–1^ rate, with different temperatures of activation and pyrolysis time. The carbonaceous resultant material was then immersed and mixed with 250 mL of a 0.1 mol L^–1^ HCl solution for about 1 h, and washed successively with hot deionized water (80 °C) to remove ZnCl_2_ and organic residual in the BPAC. This washing step was continued until the pH of the supernatant matched that of the deionized water used for washing. Approximately 2 L of hot deionized water was used to clean BPAC produced in each batch process.

BPAC was dried again for 48 h at 65 °C and ground, and the powder that passed through a 35-sieve was stored for subsequent experiments. Figure 1 shows a previous step-by-step flowchart diagram of the BPAC development process.

**Figure 1:**
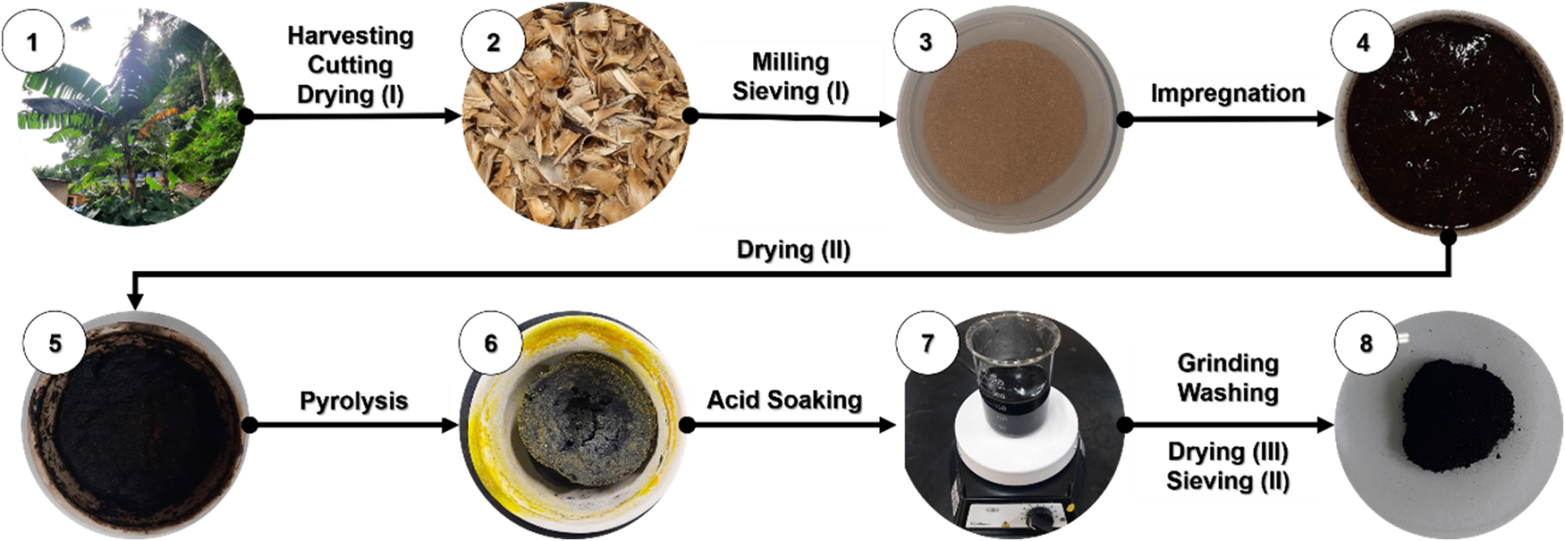
Flowchart diagram for BPAC development process, showing 8 stages from harvesting to the synthesis of the activated carbon.

### 2.3 Proximate Analysis

The moisture, volatile matter, fixed carbon, and ash contents of banana pseudostem were obtained according to the ASTM D1762–84 standard,^41^ using a porcelain crucible and replacing wood for banana pseudostem powder. Fixed carbon content was determined as the difference between 100% and the percentages of moisture, volatile matter, and ashes.

### 2.4 Specific Surface Area Analysis and Pore Distribution Size

Specific surface area analysis was carried out by nitrogen physisorption at 77 K using a surface area and porosity analyzer instrument (Anton Paar, Nova 600 Series). Approximately 0.5 g of powder was introduced into the glass bulb and degassed in the equipment for the removal of moisture and other volatile materials for 3 h at 105 °C. Then, the glass bulb was transferred into a liquid nitrogen bath for surface area determination using the BET (Brunauer–Emmett–Teller) method. This analysis was implemented for BP, and all produced BPAC. The pore volume and pore size distribution of the optimal BPAC were evaluated through the BJH (Barrett–Joyner–Halenda) approach, using nitrogen adsorption–desorption isotherms measured at 77 K. Mesopore volumes and pore size distributions were derived from the desorption branch of the isotherms.

### 2.5 X-ray Diffraction (XRD)

XRD analysis was performed using a diffractometer (Bruker, D8-Discover) with a Cu-kα radiation source (λ = 1.5418 Å), filtered with a Ni filter. The scans were conducted in the 2θ range from 10 to 70°, with a step size of 0.05°. The samples were fixed in a glass holder with propylene glycol drops added for better dispersion and fixation.

### 2.6 Scanning Electron Microscopy (SEM)

SEM analysis was performed using a scanning electron microscope (Jeol, JSM-6010LA) with a resolution of 4 nm and an electron beam voltage of 20 kV. The images were acquired in 400× and 15,000× magnifications. Prior to SEM analysis, the samples were coated with a gold layer approximately 12–20 nm thick to enhance conductivity. A complementary analysis of energy-dispersive X-ray Spectroscopy (EDS) was carried out with a silicon drift detector coupled to the microscope with an electron beam voltage of 15 kV and resolution of 133 eV.

### 2.7 Point of Zero Charge (pH_pzc_) and ζ-Potential (ζ)

The point of zero charge was performed according to Akkari et al. (2023).^42^ Initially, 50 mL of NaCl 0.01 mol L^–1^ was disposed in conical flasks (125 mL capacity), varying the pH values from 2.0 to 12.0 in 2 unit increments. For pH adjustment, stock solutions of NaOH 0.1 mol L^–1^ and HCl 0.1 mol L^–1^ were used. An amount of 0.15 g of BPAC was added to the recipients and then placed in a shaker at 120 min^–1^ rotation for 24 h. After that, the pH values were measured again. The pH_pzc_ was found as the point of intersection between the pH_final_ × pH_initial_ curve and the bisector. The assays were conducted in duplicate.

The ζ-potential was determined by using a particle analyzer (Anton Paar, Litesizer 500). Previously, a stock solution of activated carbon 0.5 mg L^–1^ was prepared by mixing a certain amount of BPAC with a 0.1 mol L^–1^ NaCl solution. Before solid dispersion, BPAC was sieved through a 270-mesh sieve. Then, it was mixed for 24 h using a magnetic stirrer and subsequently subjected to ultrasonic mixing to ensure dispersion for another 2 h. The stock BPAC solution was diluted to a 0.1 mg L^–1^ and a 25 mL volume was selected for pH adjustment, varying from 2.0 to 10.0 in 2 units increments, using 0.1 mol L^–1^ concentration of HCl or NaOH solutions. After this, an aliquot of approximately 3 mL was put in a cuvette and analyzed in a particle analyzer. This experiment was conducted in duplicate.

### 2.8 Response Surface Methodology

A CCRD was employed in this study to evaluate the effects of three selected independent variables (independent factors), which are activation temperature (°C), impregnation ratio, and pyrolysis time (min), on the response variable (dependent factor), which is the specific surface area. The banana pseudostem was employed as a precursor for activated carbon development, and ZnCl_2_ was used as a chemical activator. The banana pseudostem activated carbon was developed according to Section 2.2. The number of assays was calculated according to eq (S2) in the SI. Each assay from CCRD was identified following the pattern of AT/IR/PT.

Considering 3 independent factors and 7 replicates in central points, the total number of assays for BPAC development is 21. The statistical analysis and response surface were constructed with Design Expert 13.0 software. Table 1 shows the correspondence of coded levels (−α, −1, 0, 1, and + α) for each independent factor. Before applying the model regression to experimental data, an outlier test with central points was performed using Grubbs’ method (*p* < 0.05).

**Table 1:** Correspondence of Coded to Actual Levels of Independent Factors Applied in the Central Composite T-Rotational Design

	levels correspondence				
independent factor	–α	–1	0	1	+α
AT (°C)	264	400	600	800	936
IR	1.3	2.0	3.0	4.0	4.7
PT (min)	40	60	90	120	140

### 2.9 Adsorption Kinetics

Adsorption kinetics was conducted in batch mode using a shaker (SP Labor, SP-223) with a temperature fixed at 20 °C and 120 min^–1^ rotation. The assays were performed in duplicate with an initial concentration of 60 mg L^–1^ phosphate using KH_2_PO_4_ salt and an adsorbent dosage of 2 g L^–1^. The samples were collected in time periods of 5, 7, 15, 20, 30 min, 1, 2, 4, 8, 16, 37 and 48 h, and phosphorus quantification was executed according to ascorbic acid method preconized in Standard Methods for the Examination of Water and Wastewater (APHA, 2023)^43^ which has a minimum detection of 0.01 mg L^–1^. The adsorbed amount of phosphates was calculated based on mass balance, as shown in eq (S3) in the SI. The pseudo-first-order,^44^ pseudo-second-order^45^ and Elovich^46^ models were fitted to experimental data according to eqs (S4–S6) in the SI, respectively. The intraparticle diffusion model (Weber and Morris)^47^ was fitted to experimental data according to eq (S7) in the SI.

### 2.10 Adsorption Isotherm

The adsorption isotherm was conducted in batch mode using a shaker (SP Labor, SP-223) with the temperature fixed at 20 °C and 120 min^–1^ rotation. The assays were performed in duplicate with phosphate concentrations of 10, 15, 20, 25, 30, 40, 50, and 60 mg L^–1^, using KH_2_PO_4_ salt and an adsorbent dosage of 2 g L^–1^. The solution was continuously stirred for 24 h to ensure that equilibrium was reached. Phosphate quantification was determined according to the ascorbic acid method as in kinetic assay (APHA, 2023)^43^. The Langmuir,^48^ Freundlich,^49^ Sips,^50^ and Tempkin^51^ models were fitted to experimental data according to eqs (S8–S11) in the SI. The Langmuir separation factor (*R*_L_) was obtained according to eq (S12) in the SI to determine the favorable (or unfavorable) aspect of the adsorption process.

## 3 Results and Discussion

### 3.1 Proximate Analysis

The proximate analysis of banana pseudostem indicated percentages of 4.9 ± 0.1, 76.3 ± 2.3, 4.0 ± 0.2, and 15.0 ± 0.1% for moisture, volatile matter, ash, and fixed carbon, respectively (dry basis). Abdullah et al. (2023)^52^ reported a sensitivity of banana pseudostem to thermal degradation, obtaining a relatively higher volatile matter content than that found in this study (80.6%). Romero-Anaya et al. (2012)^53^ highlighted that the high volatile matter content obtained (75%) may indicate the potential of this material for gaseous fuel production. Jiang et al. (2022)^54^ reported moisture values similar to those found in the present study (5.17%).

Volatile matter contents were also comparable in studies by Ghani et al. (2017)^55^—73.4%, Ogunleye et al. (2014)^56^—70.53%, and even Silva et al. (2021)^57^—85.2%, indicating that, depending on the pyrolysis temperature, the material can develop high porosity. Significant differences in values for the same parameter are considered justifiable due to geographical location, sampling conditions, plant phenological stage, soil nutrient availability, and even temperature, since banana plants respond strongly to the environment in which they grow.^52,58^

### 3.2 Response Surface Methodology and Optimization

After applying the Grubbs method, a central point (*S*_BET_ = 1223 m^2^ g^–1^) was excluded for being considered an outlier. The average of remaining central points was 1023 ± 28 m^2^ g^–1^. For a better fitting of model to experimental data, a response potential transformation was needed, making *S*_BET_ as a *S*_BET_^λ^ with a cubic value of λ, performing better adjustment. The multifactor ANOVA showed that model intercept (*p* < 0.0001), AT (*p* < 0.0001), IR (*p* < 0.0001), PT (*p* < 0.0001), PT × IR (p = 0.0010), and IR^2^ (*p* < 0.0001) factors are significant for the specific surface area value of BPAC. Table 2 shows statistical parameter results for ANOVA.

**Table 2:** Statistical Parameters Results for Significant Factors in Model Regression Using a Multifactor ANOVA

parameter	DF	*F*-value	*p*-value
intercept	5	80.92	<0.0001
AT	1	70.28	<0.0001
IR	1	245.50	<0.0001
PT	1	30.84	<0.0001
PT × IR	1	17.17	0.0010
IR^2^	1	40.80	<0.0001
Lack of fit	9	3.89	0.0743
*R* ^2^		0.9666	
*R*_adj_^2^		0.9546	
*R*_pred_^2^		0.9265	

According to Table 2 of the ANOVA, the linear term of the IR exhibits the largest effect magnitude, with an *F*-value of 245.50, followed by the linear term of the AT (*F* = 70.28), the quadratic term of IR (*F* = 40.80), the linear term of PT (*F* = 30.84), and finally the interaction term between PT and IR (*F* = 17.17). These results indicate that the coefficients significantly influence the specific surface area of activated carbon in the following order: IR > AT > IR^2^ > PT > PT × IR. Lack of fit (*p* = 0.0743) is nonsignificant, indicating that the model adequately fits the experimental data, and deviations between predicted and observed values can be attributed to pure error. The *R* ^2^, *R*_adj_^2^. and *R*_pred_^2^. were 0.9666, 0.9546, and 0.9265, which indicate the model provides excellent fitting to experimental data and can be reliably used for prediction in the studied domain. Table 3 shows the experimental results (observed values) according to each assay of BPAC development with predicted values according to the model shown in eq 1.

1 $$S_{BET}={\left({1.50344\times 10}^{9}+1.68980\times 10^{6}AT+2.92891\times 10^{7}PT-1.46734\times 10^{9}IR-7.27576\times 10^{6}PT\times IR+2.48422\times 10^{8}{IR}^{2}\right)}^{1/3}$$

**Table 3:** Results of the Central Composite Design of Experimental Conditions for BPAC Development to Optimize *S*_BET_ Values

	coded levels			actual levels				
run	AT (°C)	IR	PT (min)	AT (°C)	IR	PT (min)	*S*_BET‑obs_ (m^2^ g^–1^)	*S*_BET‑pred_ (m^2^ g^–1^)
1	–1	–1	–1	400	2	60	1028	1037
2	–1	–1	1	400	4	60	717	648
3	–1	1	–1	400	2	120	1316	1261
4	–1	1	1	400	4	120	707	657
5	1	–1	–1	800	2	60	1210	1215
6	1	–1	1	800	4	60	941	988
7	1	1	–1	800	2	120	1371	1389
8	1	1	1	800	4	120	1031	992
9	–1.68	0	0	264	3	90	577	760
10	1.68	0	0	936	3	90	1178	1166
11	0	–1.68	0	600	1.3	90	1401	1411
12	0	1.68	0	600	4.7	90	858	869
13	0	0	–1.68	600	3	40	877	861
14	0	0	1.68	600	3	140	1051	1116
15	0	0	0	600	3	90	1028	1005
16	0	0	0	600	3	90	1000	1005
17	0	0	0	600	3	90	1023	1005
18	0	0	0	600	3	90	983	1005
19	0	0	0	600	3	90	1047	1005
20	0	0	0	600	3	90	1058	1005

Figure 2 shows graphical representations of the response surface varying the *x* and *y* axes with significant linear factors: PT × AT, AT × IR, and PT × IR.

**Figure 2:**
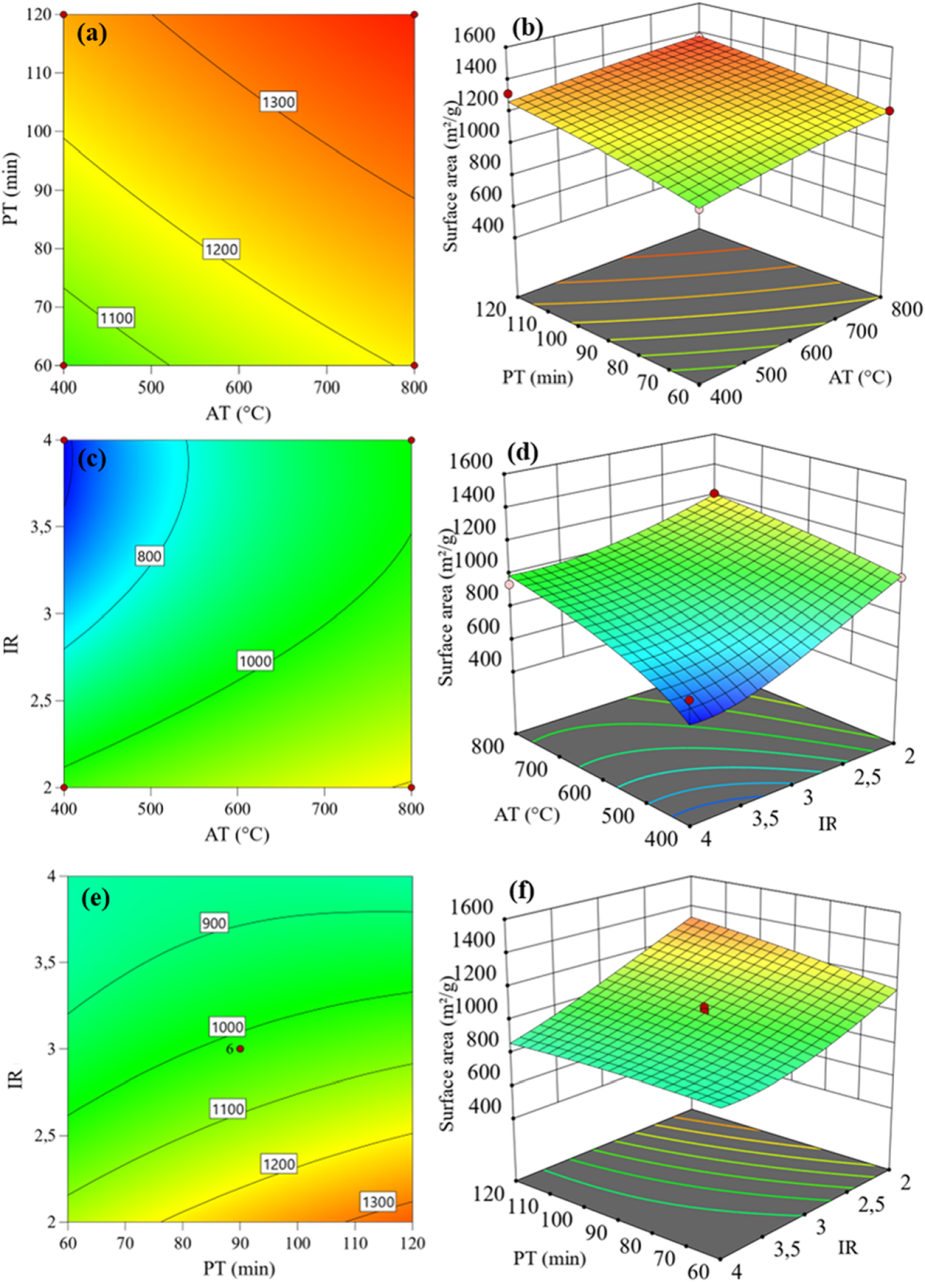
*S*_BET_ contour and surface plots according to independent factors: (a, b) PT and AT; (c, d) IR and AT; (e, f) IR and PT.

Analysis of the contour and response surface plots indicates that simultaneously increasing the pyrolysis time and the impregnation ratio leads to a reduction in the specific surface area (Figure 2e), consistent with the negative coefficient of the interaction term between these two variables (PT × IR). This decrease in specific surface area at higher IR values can be attributed to the dehydration effect induced by ZnCl_2_, which promotes the release of volatile compounds and may cause contraction of the carbon framework.^59^ Consequently, increasing both PT and IR can lead to pore collapse, enlarging micropores but reducing *S*_BET_.^60^ However, this trend occurs only up to a critical IR value. The positive quadratic coefficient (IR^2^) indicates the presence of a minimum. Below this threshold, *S*_BET_ decreases due to pore collapse, as discussed. Beyond this IR limit, the effect is reversed as excess ZnCl_2_ can penetrate the interior of the material. Facilitated by its melting above 283 °C and low surface tension, this causes the pore penetration and promotes the preservation of the structure and surface area, with excess removed during the washing step, maintaining the integrity of BPAC.^61^

The iodine number (IN) is commonly used as an indirect measure of microporosity, with higher values indicating greater microporosity and surface area.^62^ In contrast, Ghani et al. (2017)^55^ reported an opposite tendency within the same activator concentration range. While increasing ZnCl_2_ concentration initially enhanced IN, temperatures approaching 640 °C caused excessive activation, breaking pore walls and collapsing the structure, which resulted in lower IN values. This behavior can be explained by initial micropore development followed by pore enlargement and subsequent wall collapse at excessive ZnCl_2_ concentrations.^63^

For instance, when AT is fixed at 400 °C and PT is set at 60 min, IR values of 2, 3, and 4.7 correspond to *S*_BET_ values of 1037, 763 , and 772 m^2^ g^–1^, respectively. It is important to note, however, that these observations include an axial level of IR, which may not always be optimal due to its effect on surface curvature. Therefore, the selection of the optimal point is best performed within the factorial region, while validation can be extended to axial points.

All other terms in the response surface exhibit positive coefficients, suggesting a synergistic effect between these variables and the response. In other words, moving the levels from −1 to +1 leads to an increase in the specific surface area. Higher pyrolysis temperatures and longer activation times further facilitate the removal of volatile components from the biomass, supporting the formation of a well-defined porous structure with an enhanced surface area.

For adsorption assays, the optimal conditions were chosen to maximize surface area while minimizing energy input (lower pyrolysis temperature and activation time) and reagent consumption (lower impregnation ratio). Although *S*_BET_ increases with higher PT and AT, operating at the lowest practical levels is advantageous for reproducibility, feasibility, and scalability.^64^ The Design-Expert 13.0 software was employed to identify the optimal point within the factorial region, resulting in the levels 400/2/60 and a predicted specific surface area of 1037 m^2^ g^–1^, which is the optimal BPAC.

Based on the criterion of lower energy consumption (activation temperature, pyrolysis time), reactant (ZnCl_2_), and yield, a simplified cost-benefit analysis was carried out to evaluate the trade-off between surface area enhancement and the corresponding energy and reagent costs. For this purpose, the optimal activated carbon (400/2/60), with a predicted surface area of 1037 m^2^ g^–1^, and the material with the highest predicted specific surface area of 1411 m^2^ g^–1^ (600/1.3/90) were considered.

It is important to emphasize that throughout the biochar development process, the impregnation and pyrolysis steps are the ones that differ essentially in terms of costs, as they determine the residence time in the muffle furnace during pyrolysis, the heating duration, and the consumption of ZnCl_2_. Accordingly, three scenarios were established, each considering distinct electricity costs associated with muffle operation (heating and pyrolysis) and different reagent (ZnCl_2_) costs.

For the 400/2/60 BPAC, the energy consumption tends to be lower as this condition involves a lower temperature and shorter pyrolysis time. In contrast, for the 600/1.3/90 BPAC, the ZnCl_2_ consumption is reduced due to its lower impregnation ratio. Thus, Scenario 1 considered intermediate costs for both reagent and electricity, Scenario 2 assumed a high electricity cost and a low reagent cost, and Scenario 3 was based on low energy consumption and higher reagent cost.

The calculation was based on a batch of 15 g, and the data provided in the SI (cost-benefit analysis of optimal BPAC parameters) define the operational conditions and associated costs. The yield for the 600/1.3/90 BPAC was 27%, while for 400/2/60 it was 37%. Table 4 presents the total and specific costs obtained under the three different scenarios for the two BPAC analyzed.

**Table 4:** Total Costs and Unit Cost of BPAC Area for the Optimal Selected Conditions (400/2/60) and Highest BPAC Area Obtained by RSM

scenario	BPAC	total costs (US$)	specific costs (10^–3^ US$ m^–2^)
1. intermediate costs of energy and ZnCl_2_	400/2/60	1.67	0.29
	600/1.3/90	2.08	0.36
2. high energy cost and low ZnCl_2_ cost	400/2/60	1.52	0.26
	600/1.3/90	2.01	0.35
3. low energy cost and high ZnCl_2_ cost	400/2/60	1.91	0.33
	600/1.3/90	2.22	0.39

For all scenarios considered, both total and specific costs were more favorable for the production of the activated carbon defined here as the optimal BPAC (400/2/60). In comparison, the total costs increased by approximately 24, 35, and 18% for Scenarios 1, 2, and 3, respectively. Even in the latter case, where the 600/1.3/90 treatment could theoretically be more advantageous (due to the lower energy price associated with longer heating time and higher temperature and the reduced ZnCl_2_ consumption under higher reagent cost), the 400/2/60 treatment still proved to be more economically favorable.

The yield of the BPAC can be considered a critical factor as it determines the final amount of BPAC produced and, consequently, the total surface area. In a hypothetical scenario where the yields of both BPAC would be similar, despite the lower total cost of the 400/2/60 treatment across all scenarios, the total surface area of the 600/1.3/90 material would be higher and could therefore compensate for a lower specific cost. Thus, it can be concluded that, in addition to the optimization criteria established by the RSM, the economic requirements of the synthesis process also indicate that the lower levels in the factorial region (400/2/60) represent an optimal point for the specific surface area optimization.

Model validation was conducted by producing additional biochars at different activation temperatures, pyrolysis times, and impregnation ratios, including conditions outside the factorial region. Table 5 presents the predicted *S*_BET_ values (*S*_BET‑pred_), the observed responses (*S*_BET‑obs_), and the acceptable surface area range for the model at a 5% significance level.

**Table 5:** Experimental Results (Observed) Compared to Predicted Values of *S*_BET_ ^a^

condition	*S*_BET‑pred_ (m^2^ g^–1^)	*S*_BET‑obs_ (m^2^ g^–1^)	range *S*_BET_ (m^2^ g^–1^) (α = 95%)	*S*_BET‑pred_/*S*_BET‑obs_
optimal BPAC (1)	1037	2415	970–1097	0.43
optimal BPAC (2)	1037	1240	970–1097	0.84
936/2/120	1428	1418	1388–1465	1.00
500/3.5/80	819	925	760–869	0.88

a (1) Means an optimal BPAC developed in multiple batches. (2) Means the optimal BPAC developed by one batch assay for model validation.

The 936/2/120 assay was the only BPAC that resulted in the unit *S*_BET‑pred_/*S*_BET‑obs_ ratio. Although optimal BPAC (2) and 500/3.5/80 have achieved an out-of-range *S*_BET_, it is possible to consider they are relatively very close to unit, with ratio above 0.80. The model may be improved by controlling other variables in the pyrolysis process, such as oxygen concentration, crucible sealing, and temperature distribution inside the muffle furnace.

The optimal BPAC (1) yielded 36 ± 2% with a specific surface area more than twice the predicted value. This outcome may be related to the multiple-batch process used for the production of activated carbon. For this assay, five batches were performed, each with three crucibles. The washing step was carried out jointly to assess the feasibility of production scale-up. Thus, as each batch was completed, the subsequent activated carbons intended for the washing step were left soaking in 0.1 mol L^–1^ HCl solution until all batches had been processed.

Although the BPAC–HCl solution system remained at rest, the HCl solution, even at low concentration, may have further enhanced the surface area due to the longer contact time since this parameter differed from that commonly employed in the other BPAC syntheses. Tsai et al. (2023)^65^ reported the effect of the washing solution on the development of the surface area of biochar produced from pineapple peel, observing an increase in surface area when a 0.1 mol L^–1^ HCl solution was applied, the same concentration used in the present study for the primary washing step.

Bazan-Wozniak et al. (2019)^66^ studied the effect of 5% HCl solution on washing activated carbons derived from supercritical extraction of hops residues. They observed that postactivation acid washing nearly doubled the BET surface area compared to the unwashed sample. When compared with water-washed carbon, the surface area increased by almost 400 m^2^ g^–1^ after HCl washing. Similarly, Spencer et al. (2024)^67^ reported that the introduction of an acid-washing step effectively removes inorganic components from ash, which contributes to additional porosity development and higher surface area. The effect of different acid washing on the dissolution of inorganic components and pore unblocking has also been reported in other studies.^68–72^

The BET surface area of optimal BPAC (2415 m^2^ g^–1^), which greatly exceeds the RSM-predicted value (1037 m^2^ g^–1^), represents a major finding in this study. High specific surface areas obtained by chemical activation from lignocellulosic precursors have been reported in several studies. Bhat et al. (2023)^73^ achieved 2200 m^2^ g^–1^ using abundant cilantro as a precursor and KOH as the activating agent. Wei et al. (2022)^74^ used NaOH activation of macroalgae, obtaining a specific surface area of 1238.5 m^2^ g^–1^. Other studies employing ZnCl_2_ activation reported *S*_BET_ values of 1151.6 m^2^ g^–1^ for mango peels,^75^ 1700 m^2^ g^–1^ for peanut shells,^76^ 1202.2 m^2^ g^–1^ for mixed juice pulp and pomegranate peel,^77^ 1694 m^2^ g^–1^ for cocoa pod husks,^78^ and 1202.7 m^2^ g^–1^ for marine red algae.^79^ Although the reported surface area values are remarkably high, it is essential to highlight that the optimal point obtained in this study surpassed all of them, even when compared to the same activating agent. This finding demonstrates the strong potential of banana pseudostem as a promising precursor for the development of high-surface-area materials, given its abundant availability and year-round production in Brazil and tropical countries in the world.^80^

This higher value does not invalidate the predictions of the RSM model, as the other three validation points showed *S*_BET‑pred_/*S*_BET‑obs_ ratios close to unity and an *R* ^2^ of 0.9666. The maximum predicted value was overestimated due to factors not considered in the model, such as the extended soaking time with HCl solution during the post-pyrolysis multibatch process, resulting in a surface area more than twice the predicted value. Although the observed optimal BPAC (1) surface area obtained in the multibatch process appears beneficial for adsorption and catalytic applications, these findings also highlight a limitation of the RSM model, as other significant post-pyrolysis factors not included in the experimental design can influence the achievable surface area.

### 3.3 Specific Surface Area Analysis from Optimal BPAC

The N_2_ adsorption isotherm at 77 K resulted in an adsorption–desorption curve with hysteresis at a relative pressure of around 0.4. The Brunauer–Emmett–Teller (BET) method indicated a type IV­(a) isotherm, according to IUPAC technical recommendations,^81^ which is characteristic of a mesoporous adsorbent. The material exhibited a surface area of 2415 m^2^ g^–1^, an average pore size of 3.45 nm, and a pore volume of 1.65 cm^3^ g^–1^. Figure 3 shows the BET isotherm and the pore size distribution obtained by the Barrett–Joyner–Halenda (BJH) method for optimal BPAC.

**Figure 3:**
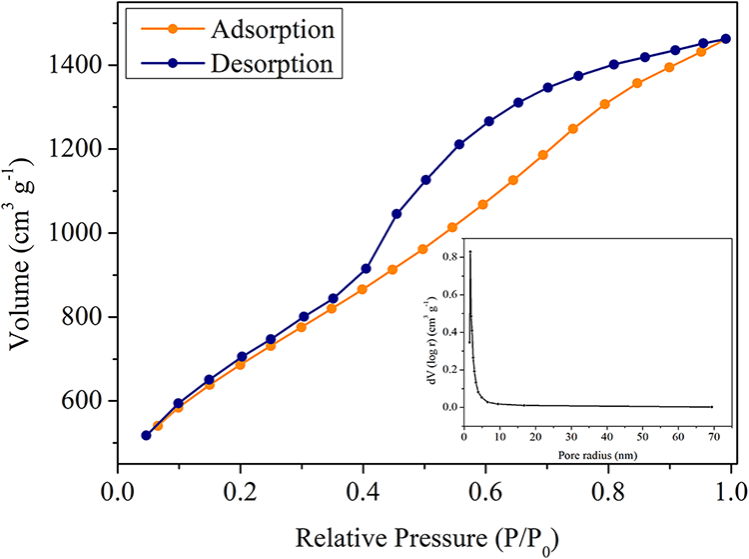
N_2_ isotherm adsorption (77 K) and pore size distribution from optimal BPAC (400/2/60).

The desorption curve showed a plateau between relative pressures of 0.4 and 1.0, indicating hysteresis due to pores exceeding the critical width. The average diameter at the optimal point classifies the material as mesoporous since it falls within the 2–50 nm range. The pore size distribution shows a range close to the mean, indicating relatively uniform sizes. Besides the pyrolysis temperature and activation time, the small size of zinc chloride and its hydrated complexes may explain the distribution’s concentration at lower values.^61^

### 3.4 X-ray Diffraction

Figure 4 shows the diffractograms for the raw banana pseudostem and optimal BPAC.

**Figure 4:**
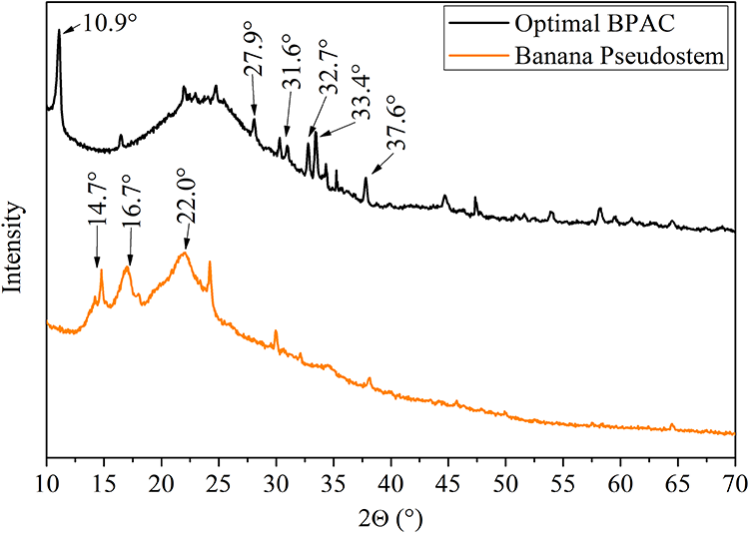
XRD diffractograms for optimal BPAC (400/2/60) and raw banana pseudostem.

The observed XRD peaks from banana pseudostem at 2θ of 14–17° and 22° correspond to the crystalline structure of cellulose I in planes 1–10 and 200, respectively, with an amorphous valley at 2θ = 18.5°.^82,83^ Although additional peaks are observed in the optimal BPAC, none of them are related to zinc oxides (2θ values of 39, 46.3, 49.7, 56.9, 70.5, and 76.8°), indicating an efficient removal of the chemical activator during the washing step.^22^

Peaks of 2θ approximately 2θ at 10.9, 27.9, 31.6, 32.7, 33.4, and 37.6° suggest the formation of lamellar zinc hydroxychlorides, with typical hexagonal nanosheets of simonkolleite (Zn_5_(OH)_8_Cl_2_·H_2_O). It is consistent with the high ZnCl_2_ impregnation (IR = 2), using a solution of approximately 4.40 mol L^–1^ (30 g of ZnCl_2_ in 50 mL of deionized water used for impregnate banana pseudostem).^84^ Well-defined XRD peaks indicate the development of aligned crystallographic planes, reflecting increased crystallinity, although the majority of the material remains amorphous, as evidenced by diffuse halo around 15° < 2θ < 30°.^82^

Simonkolleite is a zinc chloride monohydrate (layered double hydroxides) with applications in biomedical fields, and water pollutant removal techniques, including adsorption.^85,86^ Taglieri et al. (2023),^87^ Qu et al. (2023)^88^ and Momodu et al. (2015)^89^ describe simonkolleite formation by Zn^2+^, OH^–^, Cl^–^, and H_2_O, noting that crystal growth can be slow and sensitive to temperature and pH. In this study, the longer soaking time in HCl solution and the multibatch process may have enhanced the surface development of simonkolleite on optimal BPAC.

Chlorine anions occupy each top corner of the crystal and can be exchanged by other anions via ionic exchange.^90^ Co-doping strategies, such as fluorine-chlorine exchange, have been shown to enhance phosphate adsorption by layered double hydroxides,^91^ suggesting that the simonkolleite developed in optimal BPAC could improve phosphate adsorption. Simonkolleite is stable in the pH range of 5.5–7.0, and this layered double zinc hydroxide promotes phosphate adsorption through electrostatic attraction and ionic exchange, since the crystal can act as a specific binding site. However, in lower pH values, the removal tends to decrease due to partial dissolution of crystal.^92,93^ In this study, the adsorption tests were conducted in a pH range between 4.2 and 4.8, which may result in a partial dissolution of the crystal, indicating the importance of pH control to maintain stability and adsorption efficiency, and minimize potential environmental risks.

### 3.5 Scanning Electron Microscopy

The SEM images shown in Figure 5 indicate a difference in the surface morphology among the synthesized activated carbon, raw banana pseudostem, and the biochar developed in the same conditions of optimal BPAC, except for zinc chloride addition.

**Figure 5:**
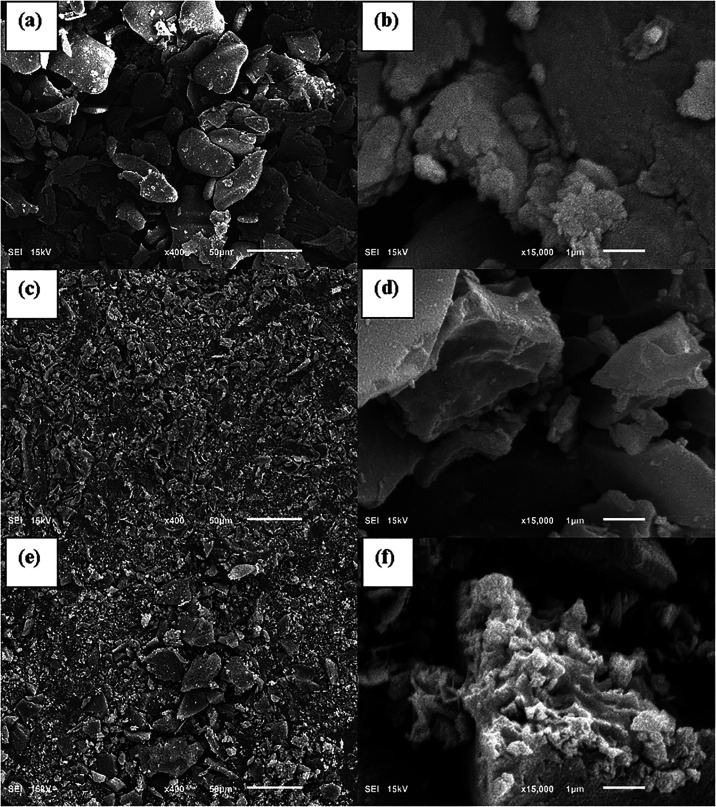
SEM images in 400× (a, c, e) and 15,000× (b, d, f). Lines “(a, b)”, “(c, d)”, and “(e, f)” indicate raw banana pseudostem, banana pseudostem biochar synthesized at 400 °C and 60 min without any chemical activator added, and optimal BPAC (400 °C, 60 min, and IR of 2), respectively.

The granules indicated in Figure 5a are bigger than (c) and (e) under the same magnification. The magnified sections (b, d, f) show a more specific morphology. The banana pseudostem (b) has a more uniform surface texture compared to (d), which has a rougher surface morphology, but still smoother than (f). It reveals that pyrolysis by itself may enhance roughness in banana pseudostem due to its thermic decomposition, but the addition of zinc chloride improves the roughness of the final activated carbon. The surface area increased with the implementation of ZnCl_2_ as a chemical impregnant. Raw banana pseudostem obtained a specific surface area of 0.904 m^2^ g^–1^, while the nonimpregnated biochar (400/0/60) and optimal BPAC (400/2/60) showed 88.058 and 2415 m^2^ g^–1^, respectively.

The EDS analysis showed for both pyrolyzed banana pseudostem a high oxygen (21.63 and 24.23%) and carbon contents (78.37 and 71.61%) for biochar without impregnation (400/0/60) and optimal BPAC (400/2/60). For the raw banana pseudostem, the contents of the O and C were close to 44.60 and 49.18%, respectively, and a potassium percentage (*K*) of 6.22%. The residual missing percentages to complete 100% were not described due to the uncertainty of the equipment. Figure 6 shows spectra obtained for these materials.

**Figure 6:**
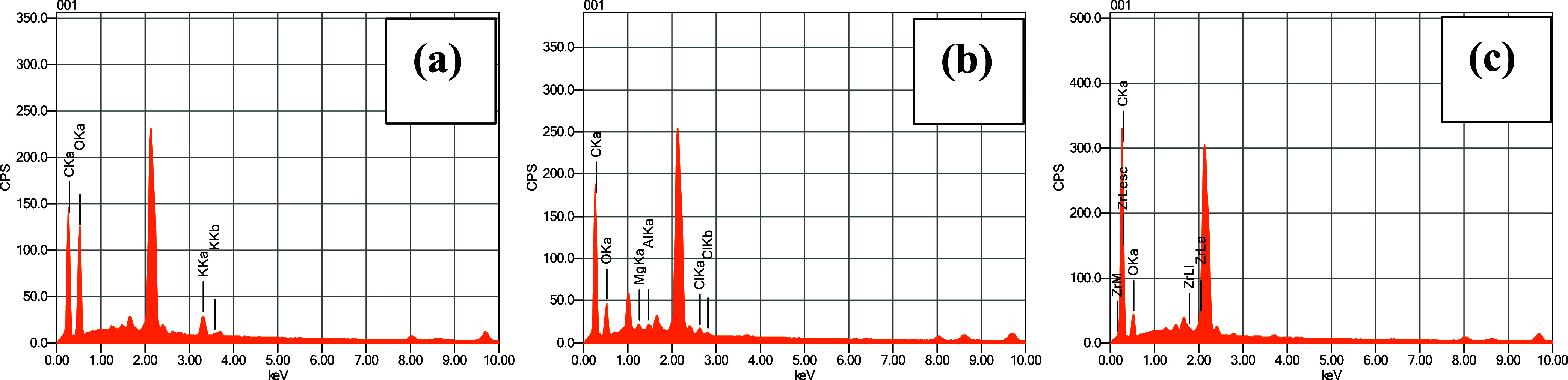
EDS spectrum for (a) raw banana pseudostem; (b) biochar without impregnation (400/0/60) and (c) optimal BPAC.

The three materials showed a characteristic peak at 2.00 keV, with close highs (225–300 CPS) which can be related to carbon tape used for fixing samples to stub. The spectrum of optimal BPAC included a Zr peak to assess potential cross-contamination from the porcelain crucible after pyrolysis, which may contain this metal in its composition. There was no detection, since the Zr peak could be misinterpreted as uncertainties. The differences obtained in C and O contents demonstrate the effect of volatilization and the enhancement of C in porous matrices from pyrolysis.

### 3.6 Point of Zero Charge and ζ-Potential

Both values of pH_pzc_ and ζ are important to comprehend the adsorbent surfaces. For the optimal BPAC, pH_pzc_ was approximately 7.30. It indicates that below this value, the activated carbon preferentially adsorbs anions, and above that, the cation adsorption is favored. According to Akkari et al. (2023),^42^ in pH values below pH_pzc_, the protonation effect is promoted in the functional groups in the surface, due to H^+^ excess. Otherwise, in superior pH_pzc_ values, the excess of OH^–^ reacts with the H^+^ from functional groups. This way, phosphate is preferentially adsorbed in pH values below 7.30. The isoelectric point (ζ_0_) was found at a pH value of 2.36. In the range of pH from 2.00 to 12.00, the ζ-potential varied between +3.02 and −16.27 mV. It indicates that when pH increases, ζ tends to be more negative, with a more pronounced decrease between 2.00 and 4.00.

The pH_pzc_ and ζ are complementary analyses, though some studies have not demonstrated differences between these techniques. For solid–liquid systems, ζ-potential refers to the external charge surrounding the particle, while point of zero charge is related to the total net surface charge. The difference observed between pH_pzc_ and ζ_0_ can be explained by an adsorption process that occurred after 24 h in the potentiometric method.^94^ Figure 7 shows the graphs profiles from (a) potentiometric and (b) electrokinetic measurements for optimal BPAC.

**Figure 7:**
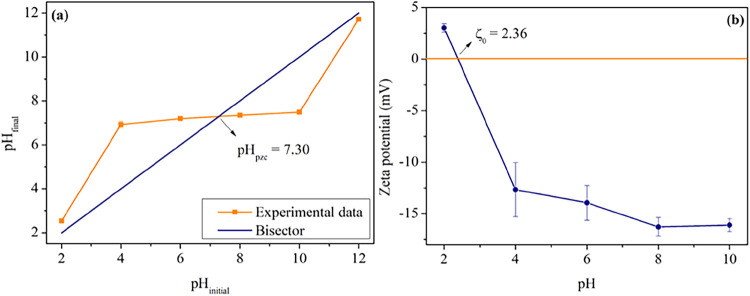
(a) pH_final_ versus pH_initial_ curve with pH_pzc_ in the intersection with bisector. (b) ζ-Potential varying pH values. Both graphs are disposed for optimal BPAC (400/2/60).

The pH values in adsorption assays varied from 4.20 to 4.80, which may have favored the affinity from phosphates to BPAC, through an electrostatic interaction with a lower value than pH_pzc_. Many studies have applied lignocellulosic residues as precursors of activated carbon and found pH_pzc_ values near neutrality. Akkari et al. (2023)^42^ and Tan et al. (2024)^95^ obtained pH_pzc_ = 6.87 (both studies) employing raw pomegranate peel biochar and activated carbon derived from algae species impregnated with KOH. Yang et al. (2024)^96^ found pH_pzc_ = 6.5 with a biochar derived from pyrolysis of cotton straw modified with Fe/Fe_3_O_4_. Tang et al. (2019)^97^ obtained a more basic pH_pzc_ value of 7.8 with a rice husk as precursor. They also verified that the time during washing and lanthanum impregnation may vary the pH_pzc_ from 7.8 to 9.1. It highlights that impregnation and functionalization can develop materials with different charges on the surface and then change de pH_pzc_.

### 3.7 Adsorption Kinetics and Isotherm

The pH values for all kinetics conical flasks ranged from 4.20 to 4.80, which indicates that the main form of phosphate ion is H_2_PO_4_^–^ (≈99.2% abundance) and HPO_4_^2–^ (≈0.8% abundance). The projection diameter from phosphate ions varies between 0.560 and 0.604 nm.^98^ Considering that the average pore size from optimal BPAC is approximately 3.45 nm, the adsorbent does not offer an obstacle for ion percolation. The kinetic models proposed in this work do not describe the adsorption mechanisms involved. If *q_t_* versus *t* ^0.5^ line results in intercepts origin, the intrapore diffusion is the limiting step in the adsorption process.^99^ The statistical and model parameters are given in Table 6.

**Table 6:** Model and Statistical Parameters for Kinetics Assays Using Optimal BPAC (400/2/60) at 20 °C

	parameters	pseudo-first-order		pseudo-second-order		Elovich	
	model	*q*_e_ (mg g^–1^)	11.458 ± 0.195	*q*_e_ (mg g^–1^)	11.963 ± 0.173	β_E_ (g mg^–1^)	1.062 ± 0.195
		*k*_1_ (h^–1^)	6.388 ± 0.509	*k*_2_ (g mg^–1^ h^–1^)	0.823 ± 0.080	α_E_ (mg g^–1^ h^–1^)	22191.545 ± 40030.373
statistical	RMSE^a^ (mg g^–1^)	0.540		0.427		1.278	
	*R* ^2^	0.980		0.987		0.887	
	*R*_adj_^2^	0.978		0.986		0.876	
	AIC^b^	–9.543		–15.647		12.870	

a RMSE: Root Mean Square Error.

b AIC: Akaike Information Criterion.

The statistical results showed the highest values of *R* ^2^ and *R*_adj_^2^ and the lowest RMSE and AIC for pseudo-second-order model, which indicates this model is the most appropriate to represent experimental data. The pseudo-first-order and pseudo-second-order obtained relatively near RMSE values with Akaike weights of 0.045 and 0.955, respectively. The ratio between the highest and lowest values indicates that pseudo-second-order is about 21.163 times more likely to be correct. Therefore, it is possible to indicate a chemisorption in the adsorption process, where the adsorption sites become occupied, due to a stronger attraction between BPAC and phosphate.^100,101^ Figure 8 shows the model adjustment for kinetics assays (a) and *q_t_* versus *t* ^0.5^ curve with respective phases involved in the adsorption process (b).

**Figure 8:**
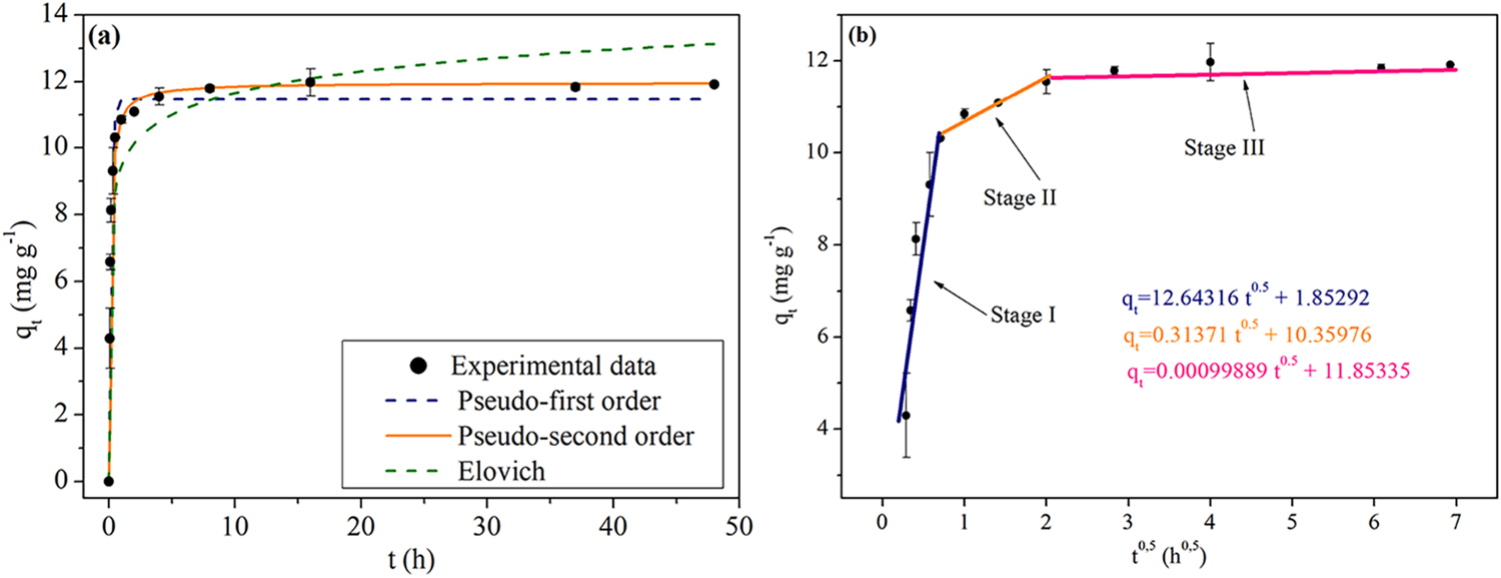
(a) Models adjustment for kinetic assays using optimal BPAC (400/2/60) with *c*_0_ = 60 mg L^–1^ at 20 °C. (b) *q_t_* × *t* ^0.5^ curve with three phases involved in the adsorption process.

According to Figure 8a and based on the best fitting model, the time of equilibrium was found in approximately 7 h with a *q_e_* of 11.963 mg g^–1^. Figure 8b presents three different stages for phosphate adsorption, with the kinetic parameters presented in Table 7. It is possible to conclude that intraparticle diffusion is not the only limiting mass transfer process, since the intercept from all stages has *b* ≠ 0. The initial phase (Stage I) is related to film diffusion, where mass transfer is controlled by the resistance offered by external boundary layer. The intermediate stage (Stage II) occurs when adsorbate diffuses through the adsorbent (intraparticle diffusion). The equilibrium phase is represented in Stage III, where the adsorption rate decreases due to adsorbent saturation and the desorption rate is equal to adsorption rate.^102,103^

**Table 7:** Kinetic Parameters Calculated by Phosphate Adsorption at 60 mg L^–1^ Using Optimal BPAC (400/2/60) to the Weber and Morris Model

Weber and Morris	stage I	stage II	stage III
*k*_D_ (mg g^–1^ h^–0.5^)	12.64316	0.31371	0.00099889
*b* (mg g^–1^)	1.85292	10.35976	11.85335

Thus, phosphate adsorption was controlled by film diffusion, and, given the presence of multiple stages, intraparticle diffusion also contributed to the overall process.^104,105^

The statistical and model parameters of the isotherm assays are given in Table 8. The Langmuir model was better fitted to the experimental data with the lowest RMSE (0.208 mg g^–1^) and AIC (−19.718). The Sips model obtained a bigger *R* ^2^ value compared to Langmuir equals *R*_adj_^2^ values for both models. However, it is important to remember that Sips is a three-parameter model, and the fitting may be better due to “*m*_S_” extra parameter. Therefore, the AIC weights can be a relevant factor in model selection, which for Langmuir and Sips models are 0.962 and 0.038, respectively. The ratio between 0.962 and 0.038 indicates that the Langmuir model is approximately 25 times more likely to be correct than Sips model. It infers that *R* ^2^ may not be sufficient for model comparison, as the addition of an extra parameter can enhance the model’s fit.

**Table 8:** Model and Statistical Parameters for Isotherms Assays Using Optimal BPAC (400/2/60) at 20 °C

	parameters	Langmuir		Freundlich		Sips		Temkin	
	model	*q*_max_ (mg g^–1^)	11.780 ± 0.128	*K*_F_ (mg^1–(1/*n*)^ *L* ^(1/*n*)^ g^–1^)	6.455 ± 0.487	*q*_max‑S_ (mg g^–1^)	11.930 ± 0.255	*B* (mg g^–1^)	1.710 ± 0.173
		*K*_L_ (L mg^–1^)	0.868 ± 0.050	*n*	5.588 ± 0.882	*K*_S_ (L^ms^ mg^–ms^)	0.855 ± 0.057	*A* (L mg^–1^)	34.278 ± 19.365
						*m*_S_	0.941 ± 0.080		
statistical	RMSE (mg g^–1^)	0.208		0.866		0.215		0.638	
	*R* ^2^	0.997		0.956		0.998		0.976	
	*R*_adj_^2^	0.997		0.949		0.997		0.972	
	AIC	–19.718		5.957		–13.276		0.437	

The Langmuir model is better fitted to experimental data, indicating a homogeneous energy site and a monolayer adsorption.^106–108^ Then, the optimal BPAC has specific sites able to adsorb only one molecule of adsorbate. The equilibrium is achieved when all these sites are filled and a monolayer is generated, with a constant heat of adsorption.^109^ Figure 9 illustrates all models fitting to experimental isotherm data at 20 °C.

**Figure 9:**
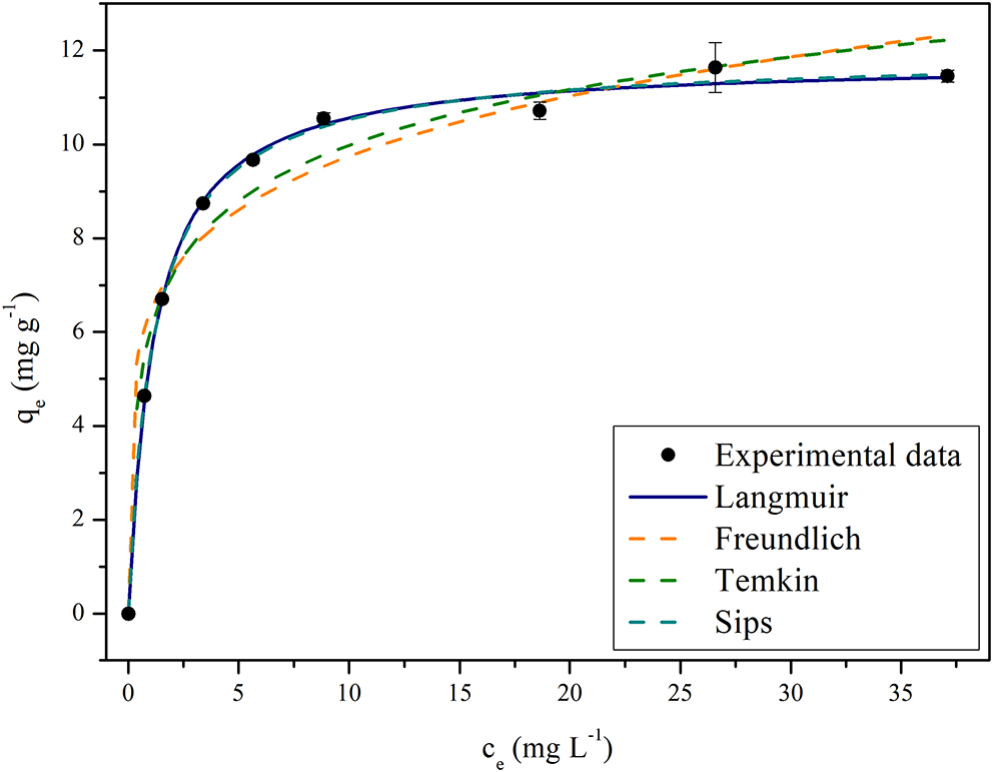
Isotherm fitting models to experimental data at 20 °C using an optimal BPAC (400/2/60).

The Langmuir separation factor applied a *c*_0_ concentration of 10 mg L^–1^ resulted in an *R*_L_ value of 0.103. If this factor is in the range between 0 and 1, then adsorption is favorable, and if *R*_L_ = 0, the adsorption is irreversible. Otherwise (*R*_L_ > 1), the process is unfavorable.^110^ An *R*_L_ value of 0.103 indicates phosphate has a good interaction with optimal BPAC. When *c*_0_ is 60 mg L^–1^ (the highest concentration for the isotherm assay in this study), *R*_L_ resulted in 0.020, which demonstrates a higher affinity to the adsorbent.^111,112^

According to Table 8 and Figure 9, the Temkin and Freundlich isotherms were the models with highest deviations. Both models are based on a heterogeneous solid surface. Freundlich is an empirical model used in low adsorbate concentration, and Temkin is based in a continuous distribution of site energies in a nonuniform surface. Sips represents a hybrid model. When “*m*_S_” parameter is equal to one, Sips becomes Langmuir, and when less than one, Freundlich model properties are more prominent. The “*m*_S_” value of 0.941 corroborates for a homogeneous aspect of optimal BPAC, which is evidenced by the better-fitted model of Langmuir.^109,113^ Table 9 shows a comparison of different materials for phosphorus adsorption with different experimental conditions and parameters.

**Table 9:** Studies with Different Adsorbents and Experimental Conditions for Phosphorus Removal

adsorbent	experimental conditions	experimental results	references
banana pseudostem activated carbon (400 °C) using zinc chloride	*T* = 20 ± 1 °C	*q*_e_ = 11.963 ± 0.173 mg g^–1^	this study
	*t*_e_ = 7 h	*k*_2_ = 0.823 ± 0.080 g mg^–1^ h^–1^	
	*c*_0_ = 60 mg L^–1^	*q*_max_ = 11.780 ± 0.128 mg g^–1^	
		*K*_L_ = 0.868 ± 0.050 L mg^–1^	
NaCl-activated clay	*t*_e_ = 0.5 h	*q*_e_ = 12.4097 mg g^–1^	^6^
	pH = 8	*k*_2_ = 239.4 g mg^–1^ h^–1^	
		*q*_max_ = 94.93 mg g^–1^	
		*K*_L_ = 0.011 L mg^–1^	
banana pseudostem activated carbon (600 °C) using zinc chloride	*T* = 18 ± 2 °C	*q*_e_ = 10.700 mg g^–1^	^7^
	*t*_e_ = 16 h	*k*_1_ = 0.254 h^–1^	
	*c*_0_ = 60 mg L^–1^	*q*_max_ = 11.82 mg g^–1^	
		*K*_L_ = 0.0842 ± 0.054 L mg^–1^	
magnetic bioactivated carbon from lignin	room temperature	*q*_e_ = 1.10 mg g^–1^	^101^
	*t*_e_ ≈ 5 h	*k*_2_ = 0.62 g mg^–1^ h^–1^	
	*c*_0_ = 7.27 mg L^–1^	*q*_max_ = 69.80 mg g^–1^	
		*K*_L_ = 0.00037 L mg^–1^	
cerium–aluminum-modified activated carbon	*T* = 25 °C	*q*_e_ = 3.094 mg g^–1^	^114^
	*t*_e_ = 3 h	*k*_2_ = 0.03 g mg^–1^ h^–1^	
	*c*_0_ = 32.67 mg L^–1^	*q*_max_ = 8.13 mg g^–1^	
		*K*_L_ = 0.023 L mg^–1^	
iron–magnesium-modified granular activated carbon	*T* = 25 °C	*q*_e_ = 0.81 mg g^–1^	^115^
	*t*_e_ = 10 h	*k*_2_ = 0.35 g mg^–1^ h^–1^	
	*c*_0_ = 20 mg L^–1^		
	pH = 7		
lanthanum-modified powder commercial activated carbon	*T* = 25 °C	*k*_2_ = 3.456 g mg^–1^ h^–1^	^116^
	*c*_0_ = 85 mg L^–1^	*q*_max_ = 176.37 mg g^–1^	
	pH = 4	*K*_L_ = 0.007 L mg^–1^	
lanthanum–magnesium-modified sheep dung activated carbon	*T* = 25 °C	*q*_e_ = 0.1782 mg g^–1^	^117^
	*t*_e_ = 7 h	*k*_2_ = 0.7307 g mg^–1^ h^–1^	
	*c*_0_= mg L^–1^	*q*_max_ = 1.94 mg g^–1^	
	pH = 4	*K*_L_ = 18.4352 L mg^–1^	
powder commercial activated carbon	*T* = 19 ± 1 °C	*q*_e_ = 2.58 mg g^–1^	^118^
	*t*_e_ = 3 h	*k*_2_ = 8.88 g mg^–1^ h^–1^	
	*c*_0_ = 5 mg L^–1^	*q*_max_ = 29.41 mg g^–1^	
		*K*_L_ = 0.042 L mg^–1^	

Comparing all studies presented in Table 9, the equilibrium time varied from 0.5 to 16 h, with an average time of 7.4 h, which is near the equilibrium time found in this study. Faria et al. (2025)^7^ was the only research that pseudo-first order had a better fitting on experimental data. It means that most of the studies, including activated carbon derived from lignocellulosic precursors, commercial activated carbon, their modification, and even activated clay, performed a chemisorption process involving phosphorus removal. Temperature is a critical parameter for adsorption and precursor origin, and specific functional groups in the material surface may be relevant in the adsorption capacity and kinetics process. The different materials offer different interactions with the same adsorbate.^119^ Other studies not described in Table 9 also have an equilibrium time superior to the reported. Biswas et al. (2023)^120^ employed pinus activated carbon impregnated with ZnCl_2_ and observed a *t*_e_ of 24 h at 25 °C with a *q*_e_ of 7.44 mg g^–1^. Mahardika et al. (2018)^121^ found a *t*_e_ varying from 10–12 h at 25 °C with a *q*_e_ value of 1.44 mg g^–1^.

According to equilibrium results presented in Table 8, it is possible to infer that optimal BPAC has one of the greatest values of *q*_max_ (11.780 mg g^–1^) overlapping within the deviation range with Faria et al. (2025),^7^ which also studied banana pseudostem as a precursor for activated carbon. However, the temperature from BPAC in this study is 200 °C lower than that in the other research. It indicates an advantage of optimal BPAC, hence costs with energy spent in the muffle furnace during pyrolysis would be lower. Wen et al. (2021)^101^ obtained a *q*_max_ value of 69.80 mg L^–1^, and this may occur due to lignin used as a precursor. This material has a high degree of purity with a better activation process due to its liquid aspect, compared to other lignocellulosic residues, as in this study.^122^

## 4 Conclusions

In the present study, pyrolysis temperature, activation time, and impregnation ratio demonstrated significant effects on surface area development of banana pseudostem activated carbon. The optimal BPAC was obtained under conditions of 400 °C, 60 min, and an IR of 2. The response surface model showed good validation results, with predicted-to-observed ratios ranging from 0.85 to 1.00, using the same method as that applied to the production of carbons from the design. The optimal BPAC exhibited an exceptionally high BET surface area (2415 m^2^ g^–1^), surpassing the predicted value. These findings demonstrate the effectiveness of the activation and soaking process in the washing step post-pyrolysis. The prolonged contact between the carbon and the acidic solution may have contributed to the increase in surface area due to an extended interaction time between the acid and BPAC, even at room temperature. The optimal BPAC produced following the criteria of lower energy levels and less reagent consumption, efficiently adsorbed phosphorus compared to other reference biochars, achieving a *q*_max_ of approximately 11.8 mg g^–1^, with a time of equilibrium reached at 7 h. Overall, the response surface methodology successfully optimized the activation conditions for banana pseudostem activated carbon, confirming the great potential of this lignocellulosic residue as a renewable and efficient precursor for producing high–surface–area carbon materials suitable for adsorption.

## Supplementary Material



## Notes

Impregnation ratio equation; CCRD assay number equation; mass balance equation; adsorption kinetic equations (pseudo-first-order, pseudo-second-order, Elovich, and Weber–Morris models); adsorption isotherm equations (Langmuir, Freundlich, Sips, and Temkin models); and cost–benefit analysis data and calculations (PDF)

## §

School of Chemical Engineering, University of Campinas (UNICAMP), Ave. Albert Einstein, 500, Campinas, SP 13083-852, Brazil

## References

[1] Soffian M. S., Halim F. Z. A., Aziz F., Rahman M. A., Amin M. A. M., Chee D. N. A. (2022). Carbon-based material derived from biomass waste for
wastewater treatment. Environ. Adv..

[2] Zhou Z., Sun H., Qiu C., Ni Y., Wang X., Zhu J. (2025). Purification and recovery
of phosphate wastewater via Fe-modified
activated carbon derived from waste phenolic distillation residue. J. Environ. Chem. Eng..

[3] Suthakaran V., Thomas R., Guirard M., Meeroff D., Jahandar
Lashaki M. (2025). Developing activated carbon adsorbent materials using
cyanobacterial biomass as precursor to remove phosphate from surface
waters. Algal Res..

[4] He Y., Qi X., Li J., Wang W., Zhang J., Yang L. (2024). Lanthanum-Integrated
Porous Adsorbent for Effective Phosphorus Removal. ACS Omega.

[5] Jung K.-W., Lee S. Y., Choi J.-W., Hwang M.-J., Shim W. G. (2021). Synthesis
of Mg–Al layered double hydroxides-functionalized hydrochar
composite via an in situ one-pot hydrothermal method for arsenate
and phosphate removal: Structural characterization and adsorption
performance. Chem. Eng. J..

[6] Kari-Ferro A., Solano-Reynoso A. M., Alvarez-Arias C., Echegaray-Peña N. G., Choque-Quispe D. (2024). Activated
polymeric materials for phosphorus removal
in aqueous medium: Study of kinetics and adsorption isotherm. Results Eng..

[7] Faria M. S. L., Lima R. M. R., de Sousa R. C. S., Borges A. C. (2025). Phosphate
adsorption
study employing a synthesized activated carbon derived from banana
pseudostem. Rev. AIDIS Ingen. Cienc. Ambient..

[8] Fan X., Wu Y., He Y., Liu H., Guo J., Li B., Peng H. (2023). Efficient removal of
phosphorus by adsorption. Phosphorus Sulfur
Silicon Relat. Elem..

[9] Mohamed S. M. I., Yılmaz M., Güner E. K., El Nemr A. (2024). Synthesis and characterization
of iron oxide-commercial activated carbon nanocomposite for removal
of hexavalent chromium (Cr6+) ions and Mordant Violet 40 (MV40) dye. Sci. Rep..

[10] El-Nemr M. A., Yilmaz M., Ragab S., El Nemr A. (2022). Watermelon
peels biochar-S
for adsorption of Cu2+ from water. Desalination
Water Treat.

[11] El-Nemr M. A., Yılmaz M., Ragab S., Al-Mur B. A., Hassaan M. A., El Nemr A. (2023). Fabrication of Pea pods biochar-NH2 (PBN) for the adsorption
of toxic Cr6+ ion from aqueous solution. Appl.
Water Sci..

[12] Li Y., Bu J., Sun Y., Huang Z., Zhu X., Li S. (2025). Efficient
degradation of norfloxacin by synergistic activation of
PMS with a three-dimensional electrocatalytic system based on Cu-MOF. Sep. Purif. Technol..

[13] Adaileh A. D., Ragab A. H., Taher M. A., Gumaah N. F., Soliman M. S. S., Taha A., Mubarak M. F. (2025). Development of a
double-shelled nanocomposite
of activated carbon-nanocellulose with cationic metal oxide core for
enhanced adsorption of bicarbonate from underground water. Inorg. Chem. Commun..

[14] Luo D., Wang L., Nan H., Cao Y., Wang H., Kumar T. V., Wang C. (2023). Phosphorus adsorption by functionalized
biochar: a review. Environ. Chem. Lett..

[15] Dong S., Li Y., Zhu K., Wang C., Zhai S. (2025). Advances in structure
designing and function tailoring strategy toward alginate-based hydrogels
for efficient water remediation: A review. Int.
J. Biol. Macromol..

[16] Shan X., Yang L., Zhao Y., Yang H., Xiao Z., An Q., Zhai S. (2022). Biochar/Mg-Al
spinel carboxymethyl cellulose-La hydrogels
with cationic polymeric layers for selective phosphate capture. J. Colloid Interface Sci..

[17] Usman M. O., Aturagaba G., Ntale M., Nyakairu G. W. (2022). A review of adsorption
techniques for removal of phosphates from wastewater. Water Sci. Technol..

[18] Du M., Zhang Y., Wang Z., Lv M., Tang A., Yu Y. (2022). Insight into the synthesis and adsorption mechanism
of adsorbents for efficient phosphate removal: Exploration from synthesis
to modification. Chem. Eng. J..

[19] Ren X., Han J., Gu P., Zhang Z., Miao H., Ni S. (2025). Characteristics
and mechanisms of phosphorus adsorption by blue algae
biochar modified with a polyaluminum chloride (PAC) dehydrating agent. J. Environ. Chem. Eng..

[20] Tsarpali M., Kuhn J. N., Philippidis G. P. (2024). Activated
carbon production from
algal biochar: Chemical activation and feasibility analysis. Fuel Commun..

[21] Amiry R. M., Mwankuna C. J., Mariki E. E., Mkoma S. L. (2025). Optimization
and
characterization of high surface area mesoporous activated carbon
produced from Syzygium cumini leaves bio-waste by zinc chloride activation. Next Res..

[22] Areti H. A., Jabesa A., Daba B. J., Jibril D. (2024). Response surface
method
based parametric optimization of Cr­(VI) removal from tannery wastewater
using a mixed banana peel and corn cob activated carbon: Kinetic and
isotherm modeling studies. J. Water Process
Eng..

[23] Gallego-Mena L., Campana R., Villardon A., Dorado F., Sánchez-Silva L. (2025). Optimisation
of hydrothermal carbonisation of olive stones for enhanced CO_2_ capture: Impact of zinc chloride activation. J. Environ. Chem. Eng..

[24] Kwon K.-S., Lee H.-S. (2025). Sustainable Development of Sawdust
Biochar as a Green
and Promising Material for CO2 Capture Technologies. Materials.

[25] Pôrto T. P., Lourenço J. C., Nogueira B., de Moraes N. P., da Silva Souto R., Siqueira A. F. (2024). Synthesis of activated
carbon from sugarcane bagasse using blends of hydroxides for maximizing
reaction targeted at obtaining hydrogen peroxide. Biomass Bioenergy.

[26] Tigrine Z., Benhabiles O., Merabti L., Chekir N., Mellal M., Aoudj S. (2024). Sustainable Activated Carbon from Agricultural Waste:
A Study on Adsorption Efficiency for Humic Acid and Methyl Orange
Dyes. Sustainability.

[27] Al-sareji O. J., Grmasha R. A., Meiczinger M., Al-Juboori R. A., Somogyi V., Hashim K. S. (2024). A Sustainable Banana
Peel Activated
Carbon for Removing Pharmaceutical Pollutants from Different Waters:
Production, Characterization, and Application. Materials.

[28] Nguyen D.
L. T., Nguyen N.-A., Pham D. D., Van Pham D., Chau N. T., Nguyen N. T. (2025). Cassava starch residue derived activated carbon-supported
low-loading ultrafine MoS2: A sustainable and efficient catalyst for
electrochemical hydrogen evolution. Int. J.
Hydrogen Energy..

[29] Bih N. L., Rwiza M. J., Ripanda A. S., Mahamat A. A., Machunda R. L., Choi J. W. (2025). Adsorption of phenol
and methylene blue contaminants
onto high-performance catalytic activated carbon from biomass residues. Heliyon.

[30] Ab
Ghani Z., Yusoff M. S., Alazaiza M. Y. D., Akinbile C. O., Binti Abd Manan T. S. (2023). Landfill leachate treatment by activated carbon (AC)
from banana pseudo-stem, iron oxide nanocomposite (IOAC), and iron
oxide nanoparticles (IONPs). J. Environ. Chem.
Eng..

[31] Dhanabal R., Priya P. G. (2025). Optimization and
photocatalytic degradation of crystal
violet dye using Sr-ZnO/activated carbon nanoneedles. Mater. Sci. Semicond. Process..

[32] Gurav R., Bhatia S. K., Choi T.-R., Park Y.-L., Park J. Y., Han Y.-H. (2020). Treatment
of furazolidone contaminated water using
banana pseudostem biochar engineered with facile synthesized magnetic
nanocomposites. Bioresour. Technol..

[33] Zhao D., Zhang S., Deng H., Hu L., Li A. (2025). Eco-Friendly
Adsorbent: Insights Into the Performance and Adsorption Mechanisms
of Banana Fruit Shaft Biochar for the Removal of Mn­(II), Cd­(II), Pb­(II),
and Cu­(II). Appl. Organomet. Chem..

[34] Zhang H., Jiang F., Zhang X., Hu S., Li J., Zhang H., Liu K. (2025). CO2 adsorption performance of nitrogen-doped
activated carbon from banana pseudo-stem by urea-assisted high-pressure
CO2-Hydrothermal treatment. Sep. Purif. Technol..

[35] Lu J. Y., Bu Z. Q., Lei Y. Q., Wang D., He B., Wang J., Huang W. T. (2024). Facile
microwave-assisted synthesis
of Sb2O3-CuO nanocomposites for catalytic degradation of p-nitrophenol. J. Mol. Liq..

[36] Kılıç M., Bekman M. E., Bodur F., Yıldız A., Varol E. A. (2025). Modeling and optimization of flash
heating process
conditions for activated carbon production using Response Surface
Methodology (RSM). Diam. Relat. Mater..

[37] Coutinho R., Hoshima H. Y., Marques M. (2025). Enhanced removal
of low-concentrations
of PFOA from water and wastewater with a novel luffa sponge-derived
adsorbent: one-step synthesis and optimization using design of experiments
and artificial neural network. Biomass Bioenergy.

[38] Sorto J. E. P., Mendonça I. F., Schultz E. L., Soares I. P. (2025). Hydrogen-rich
syngas from model biogas steam reforming over a Ni–Pr/hydrotalcite-derived
catalyst: An RSM-central composite rotational design. Int. J. Hydrogen Energy..

[39] Gonçalves I. L., de Menezes Filho F. C. M., de Morais E. B., de Castro V. A., Canales F. A. (2024). Optimization of
the use of Moringa
oleifera in wastewater treatment by rotational central composite design. Desalination Water Treat.

[40] Ritter M. T., Lobo-Recio M. Á., Padilla I., Romero M., López-Delgado A. (2024). Salt slag
and rice husk ash as raw materials in zeolite synthesis: Process optimization
using central composite rotational design. Sustain.
Chem. Pharm..

[41] ASTM. Standard Method for Chemical Analysis of Wood Charcoal. D1762–84; American Society for Testing and Materials (ASTM). International: Philadelphia, PA, 2013.

[42] Akkari I., Graba Z., Bezzi N., Merzeg F. A., Bait N., Ferhati A. (2023). Raw pomegranate peel
as promise efficient biosorbent
for the removal of Basic Red 46 dye: equilibrium, kinetic, and thermodynamic
studies. Biomass Convers. Biorefin..

[43] American Public Health Association; American Water Works Association; Water Environment Federation . Standard Methods for the Examination of Water and Wastewater, 24th ed.; Lipps, W. C. ; Baxter, T. E. ; Braun-Howland, E. , Eds.; APHA Press: Washington, DC, 2023.

[44] Lagergren S. (1898). About the
Theory of So-Called Adsorption of Soluble Substances. K. Sven. Vetenskapsakad. Handl..

[45] Ho Y. S., McKay G. (1999). Pseudo-second-order model for sorption processes. Process Biochemistry.

[46] Zeldovich J. (1934). The Catalytic
Oxidation of Carbon Monoxide on Manganese Dioxide. Acta Physicochim. URSS.

[47] Weber W. J., Morris J. C. (1963). Kinetics of Adsorption on Carbon from Solution. J. Sanit. Eng. Div..

[48] Langmuir I. (1916). The Constitution
and Fundamental Properties of Solids and Liquids. Part I. Solids. J. Am. Chem. Soc..

[49] Freundlich H. M. F. (1906). Over
the Adsorption in Solution. J. Phys. Chem. A.

[50] Sips R. (1948). On the Structure
of a Catalyst Surface. J. Chem. Phys..

[51] Tempkin M.
I., Pyzhev V. (1940). Kinetics of
Ammonia Synthesis on Promoted Iron Catalyst. Acta Phys. Chem..

[52] Abdullah N., Mohd Taib R., Mohamad Aziz N. S., Omar M. R., Md Disa N. (2023). Banana pseudo-stem
biochar derived from slow and fast pyrolysis process. Heliyon.

[53] Romero-Anaya A. J., Lillo-Ródenas M. A., Salinas-Martínez
De Lecea C., Linares-Solano A. (2012). Hydrothermal and conventional H3PO4
activation of two natural bio-fibers. Carbon.

[54] Jiang F., Cao D., Hu S., Wang Y., Zhang Y., Huang X. (2022). High-pressure
carbon dioxide-hydrothermal enhance yield and methylene
blue adsorption performance of banana pseudo-stem activated carbon. Bioresour. Technol..

[55] Ghani Z. A., Yusoff M. S., Zaman N. Q., Zamri M. F. M. A., Andas J. (2017). Optimization of preparation conditions
for activated carbon from
banana pseudo-stem using response surface methodology on removal of
color and COD from landfill leachate. Waste
Manag..

[56] Ogunleye O. O., Ajala M. A., Agarry S. E. (2014). Evaluation
of Biosorptive Capacity
of Banana (*Musa paradisiaca*) Stalk for Lead­(II) Removal
from Aqueous Solution. J. Environ. Prot..

[57] Silva M. C., Spessato L., Silva T. L., Lopes G. K. P., Zanella H. G., Yokoyama J. T. C. (2021). H3PO4–activated
carbon fibers of high
surface area from banana tree pseudo-stem fibers: Adsorption studies
of methylene blue dye in batch and fixed bed systems. J. Mol. Liq..

[58] Turner D. W., Fortescue J. A., Thomas D. S. (2007). Environmental physiology
of the bananas
(Musa spp). Brazil. J. Plant Physiol..

[59] Maruthu M., Shanmugavel D. (2025). Optimisation
and characterisation of zinc chloride
activated Saccharum spontaneum biochar for enhanced thermal energy
storage efficiency using response surface methodology. J. Energy Storage..

[60] Fan M., Shao Y., Wang Y., Sun J., He H., Jiang Y. (2024). Preparation of activated carbon with recycled ZnCl2
for maximizing utilization efficiency of the activating agent and
minimizing generation of liquid waste. Chem.
Eng. J..

[61] Hanafi N. A. M., Abdulhameed A. S., Jawad A. H., ALOthman Z. A., Yousef T. A., Al Duaij O. K., Alsaiari N. S. (2024). Optimized removal
process and tailored adsorption mechanism of crystal violet and methylene
blue dyes by activated carbon derived from mixed orange peel and watermelon
rind using microwave-induced ZnCl2 activation. Biomass Convers. Biorefin..

[62] Ganjoo, R. ; Sharma, S. ; Kumar, A. ; Daouda, M. M. A. Activated Carbon: Fundamentals, Classification, and Properties. In Activated Carbon; The Royal Society of Chemistry, 2023; pp 1–22 10.1039/BK9781839169861-00001.

[63] Kaya M., Şahin Ö., Saka C. (2018). Preparation and TG/DTG, FT-IR, SEM,
BET Surface Area, Iodine Number and Methylene Blue Number Analysis
of Activated Carbon from Pistachio Shells by Chemical Activation. Int. J. Chem. React. Eng..

[64] Kacan E. (2016). Optimum BET
surface areas for activated carbon produced from textile sewage sludges
and its application as dye removal. J. Environ.
Manage..

[65] Tsai C.-H., Tsai W.-T., Kuo L.-A. (2023). Effect of Post-Washing on Textural
Characteristics of Carbon Materials Derived from Pineapple Peel Biomass. Materials.

[66] Bazan-Wozniak A., Nowicki P., Pietrzak R. (2019). The effect of demineralization on
the physicochemical and sorption properties of activated bio-carbons. Adsorption.

[67] Spencer W., Ibana D., Singh P., Nikoloski A. N. (2024). Effect
of Surface Area, Particle Size and Acid Washing on the Quality of
Activated Carbon Derived from Lower Rank Coal by KOH Activation. Sustainability.

[68] Sierra I., Iriarte-Velasco U., Cepeda E. A., Gamero M., Aguayo A. T. (2016). Preparation
of carbon-based adsorbents from the pyrolysis of sewage sludge with
CO2. Investigation of the acid washing procedure. Desalination Water Treat.

[69] Ardian F., Putra I., Nasuci F. S. M., Wonua K. J., Purba J. Y. (2024). Optimization
of Acid Wash Process on Activated Carbon with Variation of HCL Concentration
at PT XYZ. J. Metall. Eng. Process. Technol..

[70] Park J. E., Lee G. B., Kim H., Hong B. U. (2022). High Surface Area–Activated
Carbon Production from Cow Manure Controlled by Heat Treatment Conditions. Processes.

[71] Williams N. E., Oba O. A., Aydinlik N. P. (2022). Modification, Production, and Methods
of KOH-Activated Carbon. ChemBioEng. Reviews.

[72] Wei X., Huang S., Wu Y., Wu S. (2022). Effects of washing
pretreatment on properties and pyrolysis biochars of penicillin mycelial
residues. Biomass Bioenergy.

[73] Bhat S., Uthappa U. T., Sadhasivam T., Altalhi T., Soo Han S., Kurkuri M. D. (2023). Abundant cilantro
derived high surface area activated
carbon (AC) for superior adsorption performances of cationic/anionic
dyes and supercapacitor application. Chem. Eng.
J..

[74] Wei M., Marrakchi F., Yuan C., Cheng X., Jiang D., Zafar F. F. (2022). Adsorption modeling, thermodynamics, and DFT
simulation of tetracycline onto mesoporous and high-surface-area NaOH-activated
macroalgae carbon. J. Hazard Mater..

[75] Razali N. S., Abdulhameed A. S., Jawad A. H., ALOthman Z. A., Yousef T. A., Al-Duaij O. K., Alsaiari N. S. (2022). High-Surface-Area-Activated Carbon
Derived from Mango Peels and Seeds Wastes via Microwave-Induced ZnCl2
Activation for Adsorption of Methylene Blue Dye Molecules: *Statistical Optimization and Mechanism*. Molecules.

[76] Fletcher A., Somorin T., Aladeokin O. (2024). Production of High Surface Area Activated
Carbon from Peanut Shell by Chemical Activation with Zinc Chloride:
Optimisation and Characterization. Bioenergy
Res..

[77] Suhaimi A., Abdulhameed A. S., Jawad A. H., Yousef T. A., Al Duaij O. K., ALOthman Z. A., Wilson L. D. (2022). Production of large surface area
activated carbon from a mixture of carrot juice pulp and pomegranate
peel using microwave radiation-assisted ZnCl2 activation: An optimized
removal process and tailored adsorption mechanism of crystal violet
dye. Diam. Relat. Mater..

[78] Susanti R. F., Wiratmadja R. G. R., Kristianto H., Arie A. A., Nugroho A. (2022). Synthesis
of high surface area activated carbon derived from cocoa pods husk
by hydrothermal carbonization and chemical activation using zinc chloride
as activating agent. Mater. Today Proc..

[79] Shoaib A. G. M., El-Sikaily A., El Nemr A., Mohamed A. E. D. A., Hassan A. A. (2022). Preparation and
characterization of highly surface
area activated carbons followed type IV from marine red alga (Pterocladia
capillacea) by zinc chloride activation. Biomass
Convers. Biorefin..

[80] Alzate
Acevedo S., Díaz Carrillo Á. J., Flórez-López E., Grande-Tovar C. D. (2021). Recovery
of Banana Waste-Loss from Production and Processing: A Contribution
to a Circular Economy. Molecules.

[81] Thommes M., Kaneko K., Neimark A. V., Olivier J. P., Rodriguez-Reinoso F., Rouquerol J., Sing K. S. (2015). Physisorption of gases, with special
reference to the evaluation of surface area and pore size distribution
(IUPAC Technical Report). Pure Appl. Chem..

[82] Kumar A., Mohan Jena H. (2015). High surface
area microporous activated carbons prepared
from Fox nut (Euryale ferox) shell by zinc chloride activation. Appl. Surf. Sci..

[83] Faradilla R. H. F., Lee G., Arns J.-Y., Roberts J., Martens P., Stenzel M. H., Arcot J. (2017). Characteristics of
a free-standing
film from banana pseudostem nanocellulose generated from TEMPO-mediated
oxidation. Carbohydr. Polym..

[84] Li S., Chen X., Wang X. (2019). Simonkolleite Coating
on Poly­(Amino Acids) to Improve Osteogenesis and Suppress Osteoclast
Formation in Vitro. Polymers (Basel).

[85] de
Oliveira J. M., Costa M. P. C., Perini H. F., Trevisan R. O., de Almeida L. I. M., de Matos S. L. M. (2025). From Nanocrystals and
Nanocomposites to Microcrystals: The Role of Simonkolleite/ZnO in
Overcoming Bacterial Resistance and Ensuring Biocompatibility. ACS Omega.

[86] Nartowska E., Stępień P., Kanuchova M. (2025). Impact of
Zinc­(II) Chloride Contamination on Bentonites: Formation of Simonkolleite
and Effects on Porosity and Chemical Composition. Materials.

[87] Taglieri G., Daniele V., Maurizio V., Merlin G., Siligardi C., Capron M., Mondelli C. (2023). New Eco-Friendly
and Low-Energy Synthesis
to Produce ZnO Nanoparticles for Real-World Scale Applications. Nanomaterials.

[88] Qu S., Hadjittofis E., Malaret F., Hallett J., Smith R., Campbell K. S. (2023). Controlling simonkolleite crystallisation *via* metallic Zn oxidation in a betaine hydrochloride solution. Nanoscale Adv..

[89] Momodu D. Y., Barzegar F., Bello A., Dangbegnon J., Masikhwa T., Madito J., Manyala N. (2015). Simonkolleite-graphene
foam composites and their superior electrochemical performance. Electrochim. Acta.

[90] Tanaka H., Fujioka A. (2010). Influence of thermal
treatment on the structure and
adsorption properties of layered zinc hydroxychloride. Mater. Res. Bull..

[91] Yang A., Fu Y., Huang F. (2024). Enhanced phosphorus
adsorption performance of ZnAl-LDO
by fluorine-chlorine co-doping and synergistic mechanism exploration. Sci. Total Environ..

[92] He X., Zhou X., Shang T., Liu W., Jiang G., Liu C. (2024). Influence mechanism
of different elements and alloy
phases on the corrosion resistance of Zn-Al-Mg coated steel in the
atmospheric environment: A review. Corros. Commun..

[93] Almasri D. A., Essehli R., Tong Y., Lawler J. (2021). Layered zinc hydroxide
as an adsorbent for phosphate removal and recovery from wastewater. RSC Adv..

[94] Serrano-Lotina A., Portela R., Baeza P., Alcolea-Rodriguez V., Villarroel M., Ávila P. (2023). ζ-potential as a tool for functional
materials development. Catal. Today..

[95] Tan L., Nie Y., Chang H., Zhu L., Guo K., Ran X. (2024). Adsorption performance
of Ni­(II) by KOH-modified biochar derived
from different microalgae species. Bioresour.
Technol..

[96] Yang X., Li X., Wang X., Mu Y., Tian W. (2024). Magnetic triiron tetraoxide/biochar–loadednanoscale
zero-valent iron for chromium­(VI) removal from aqueous solution. J. Taiwan Inst. Chem. Eng..

[97] Tang Q., Shi C., Shi W., Huang X., Ye Y., Jiang W. (2019). Preferable
phosphate removal by nano-La­(III) hydroxides modified
mesoporous rice husk biochars: Role of the host pore structure and
point of zero charge. Sci. Total Environ..

[98] ChemAxon . Chemicalize 2022 https://chemicalize.com/app/calculation (accessed July 11, 2025).

[99] Dharmarathna S. P., Priyantha N. (2024). Investigation
of boundary layer effect of intra-particle
diffusion on methylene blue adsorption on activated carbon. Energy Nexus.

[100] Husien S., El-taweel R. M., Mohamed N., Abdel-Aziz A. B., Alrefaey K. A., Elshabrawey S. O. (2023). Potentials of algae-based
activated carbon for the treatment of M.orange in wastewater. Case Stud. Chem. Environ. Eng..

[101] Wen Y., Zheng Z., Wang S., Han T., Yang W., Jönsson P. G. (2021). Magnetic bio-activated carbons production
using different
process parameters for phosphorus removal from artificially prepared
phosphorus-rich and domestic wastewater. Chemosphere.

[102] Wei S., Du G., Li C., Zhang L., Li J., Mao A. (2024). Removal
mechanism of Pb­(II) from soil by biochar-supported
nanoscale zero-valent iron composite materials. RSC Adv..

[103] Roy D., Roy B., Manna A. K. (2023). Pyrolyzed
mesoporous activated carbon
preparation from natural rubber common effluent biosludge: Characterization,
isotherms, kinetics, thermodynamics, and ANN modeling during phenol
adsorption. Groundw. Sustain. Dev..

[104] Gonçalves dos Santos M., Destefani Paquini L., Leite Quintela P. H., Roberto Profeti L. P., Guimarães D. (2025). Insights into
Kinetics and Thermodynamics for Adsorption Methylene Blue Using Ecofriendly
Zeolites Materials. ACS Omega.

[105] Zhao Y., Yang H., Sun J., Zhang Y., Xia S. (2021). Enhanced Adsorption of Rhodamine
B on Modified Oil-Based Drill Cutting
Ash: Characterization, Adsorption Kinetics, and Adsorption Isotherm. ACS Omega.

[106] Wang J., Wang B., Wen Z., Zhao N., Li T., Zhao W. (2023). Adsorption Process Optimization and Adsorbent Evaluation
Based on Langmuir Isotherm Model. Langmuir.

[107] Yildiz H. (2024). The production of a novel adsorbent
from forest waste
(Platanus orientalis L.) for dye adsorption: Adsorption process optimization
and experimental design. Mater. Sci. Eng.:B.

[108] Nie W., Zhang X., Luo X., Xie L., Zhu Y., Tang A., Liang D. (2025). Efficient phosphate
adsorption from
real industrial wastewater using Fe/La-impregnated biochar. J. Environ. Chem. Eng..

[109] Serafin J., Dziejarski B. (2023). Application of isotherms models and
error functions in activated carbon CO2 sorption processes. Microporous Mesoporous Mater..

[110] Benaddi E. H., Laamari M. R., Boutouil A., Bagoun R. (2024). Efficient
removal of organic pollutants from aqueous environments using activated
carbon derived from biomass waste of Calotropis procera fruit: Characterization,
kinetics, isotherm, and thermodynamic investigation. Diam. Relat. Mater..

[111] Yang F., Jin C., Wang S., Wang Y., Wei L., Zheng L. (2023). Bamboo-based magnetic activated carbon for
efficient removal of sulfadiazine: Application and adsorption mechanism. Chemosphere.

[112] El-Aryan Y. F., Melhi S., Ahmed I. M., El-Ossaily Y. A., Ali H. M., El-Gammal B., Bedair M. A. (2024). Exploring the adsorption
potential of lanthanum (III), samarium (III), and cerium (III) from
aqueous solutions utilizing activated carbon derived from date seeds. Inorg. Chem. Commun..

[113] Ibrahim El-Aswar E., Ibrahim S. S., Abdallah Y. R., Elsharkawy K. (2024). Removal of
ciprofloxacin and heavy metals from water by bentonite/activated carbon
composite: Kinetic, isotherm, thermodynamic and breakthrough curve
modeling studies. J. Mol. Liq..

[114] Wu Q., Zhang J., Wang S. (2023). Preparation
and application of modified
activated carbon for effective removal of phosphorus from glyphosate
by-product salt. Desal. Water Treat..

[115] Gao P., Zhang Y., Wang S. (2022). Increasing
the hydrophyte removal
rate of dissolved inorganic phosphorus using a novel Fe-Mg-loaded
activated carbon hydroponic substrate with adsorption-release dual
functions. J. Environ. Manag..

[116] Du M., Zhang Y., Wang Z., Lv M., Xu Q., Chen Z. (2022). La-doped activated carbon
as high-efficiency phosphorus
adsorbent: DFT exploration of the adsorption mechanism. Sep. Purif. Technol..

[117] Chen J., Chen Z., Song Z., Cao S., Li X., Wang Y. (2024). Preparation of La/Mg modified sheep dung activated
carbon and its adsorption characteristics for phosphorus in wastewater. Desal. Water Treat..

[118] Ouakouak A. K., Youcef L. (2016). Phosphates Removal by Activated Carbon. Sens. Lett..

[119] Marczewski A. W., Seczkowska M., Deryło-Marczewska A., Blachnio M. (2016). Adsorption equilibrium and kinetics of selected phenoxyacid
pesticides on activated carbon: effect of temperature. Adsorption.

[120] Biswas B., Rahman T., Sakhakarmy M., Jahromi H., Eisa M., Baltrusaitis J. (2023). Phosphorus adsorption using chemical and metal chloride activated
biochars: Isotherms, kinetics and mechanism study. Heliyon.

[121] Mahardika D., Park H.-S., Choo K.-H. (2018). Ferrihydrite-impregnated
granular activated carbon (FH@GAC) for efficient phosphorus removal
from wastewater secondary effluent. Chemosphere.

[122] Han T., Lu X., Sun Y., Jiang J., Yang W., Jönsson P. G. (2020). Magnetic
bio-activated carbon production from lignin
via a streamlined process and its use in phosphate removal from aqueous
solutions. Sci. Total Environ..

