# Supplementary material for: Activated Carbon from Banana Pseudostem: Multivariate Optimization of Synthesis and Adsorption Study for Phosphorus Removal
Source: ACS Omega. 2026 Jan 1;11(2):2930–46. doi: 10.1021/acsomega.5c08820 (PMC12824967; doi:10.1021/acsomega.5c08820)
Supplement: Supplementary file 1 [file ao5c08820_si_001.pdf]

Supporting Information

ACTIVATED CARBON FROM BANANA PSEUDOSTEM: MULTIVARIATE OPTIMIZATION OF SYNTHESIS AND ADSORPTION STUDY FOR PHOSPHORUS REMOVAL

Moisés de Souza Luz Faria[#a]
Tatianny de Araujo Andrade[a]
Renata Pereira Lopes Moreira[a]
Rita de Cássia Superbi de Sousa[a]
Alisson Carraro Borges[*b]

[a] Department of Chemistry, Federal University of Viçosa, Ave. P.H Rolfs, s/n, Viçosa, MG 36570-900, Brazil.

[b] Department of Agricultural Engineering, Federal University of Viçosa, Ave. P.H Rolfs, s/n, Viçosa, MG 36570-900, Brazil.

[*] Email: borges@ufv.br

[#] Current address: School of Chemical Engineering, University of Campinas (UNICAMP), Ave. Albert Einstein, 500, Campinas, SP 13083-852, Brazil.

1. **Impregnation Ratio Equation**

$$IR = \frac{m_{ZnCl_2}}{m_{PB}} \quad \text{(S1)}$$

Where IR is the impregnation ratio (dimensionless); m_{ZnCl_2} is the $ZnCl_2$ mass (g) and; m_{PB} is the banana pseudostem mass (fixed as 15 g for each batch pyrolysis assay).

2. **Number of assays in CCRD Equation**

$$N = 2^k + 2k + C_0 \quad \text{(S2)}$$

Where N is the total number of assays; k is the number of independent factors and; C_0 is the number of replicates in central points.

3. **Mass balance and Adsorption Kinetics Equations**

$$q = (c_0 - c)\frac{V}{m} \quad \text{(S3)}$$

$$q_t = q_e(1 - e^{-k_1 t}) \quad \text{(S4)}$$

$$q_t = \frac{k_2 {q_e}^2 t}{1 + k_2 q_e t} \quad \text{(S5)}$$

$$q_t = \frac{1}{\beta} ln(1 + \alpha\beta t) \quad \text{(S6)}$$

$$q_t = k_d t^{0.5} + b \quad \text{(S7)}$$

Where q is the quantity adsorbed (mg g^{-1}); c_0 is contaminant concentration in liquid phase (mg L^{-1}); V is the volume of sample used in batch assay (L); m is the mass of adsorbent in batch assay (g); q_t is the quantity adsorbed in an instant t (mg g^{-1}); q_e is the quantity adsorbed in equilibrium (mg g^{-1}); k_1 is the pseudo-first order adsorption rate coefficient (h^{-1}); t is time (h); k_2 is the pseudo-second order adsorption rate coefficient (g mg^{-1} h^{-1}); β is the constant related to activation energy and complexity of adsorption process (g mg^{-1}); α is the constant related to initial adsorption rate (mg g^{-1} h^{-1}); k_d (mg g^{-1} $h^{-0.5}$) and b (mg g^{-1}) both are intraparticle diffusivity constants.

4. **Adsorption Isotherm Equations**

$$q_e = \frac{q_{max} K_L c_e}{1 + K_L c_e} \quad \text{(S8)}$$

$$q_e = K_F {c_e}^{1/n} \quad \text{(S9)}$$

$$q_e = \frac{q_{max-S} K_S {c_e}^{m_S}}{1 + K_S {c_e}^{m_S}} \quad \text{(S10)}$$

$$q_e = Bln(Ac_e) \quad \text{(S11)}$$

$$R_L = \frac{1}{1 + K_L c_0} \quad \text{(S12)}$$

Where q_e is the quantity adsorbed in equilibrium (mg g^{-1}); q_{max} is the maximum adsorption capacity (mg g^{-1}); K_L is the Langmuir parameter related to adsorbate-adsorbent interaction (L mg^{-1}); c_e is the bulk concentration in equilibrium (mg L^{-1}); K_F is the Freundlich constant ($mg^{1-(1/n)}$ $L^{(1/n)}$ g^{-1}); n is the empirical parameter of Freundlich (dimensionless); q_{m-S} is the maximum adsorption capacity for Sips model (mg g^{-1}); K_S is the Sips constant (L^{ms} mg^{-ms}); m_S is the Sips parameter related to the process heterogeneity (dimensionless); B is the constant related to adsorption energy (mg g^{-1}); A is isotherm constant of Temkin (L mg^{-1}).

5. Cost-benefit analysis of optimal BPAC parameters.

Table S1 shows the supplementary data of BPAC parameters, operation parameters involved in costs of energy and reactant used for activation. The values of $ZnCl_2$ were obtained considering three different suppliers in Brazil. The price was calculated per gram of reactant and converted in US$ using the average of last 3 months, which resulted in a value of 1 US$ = R$ 5.3988. The costs related to electricity for heating muffle furnace was obtained according to three tariffs from Minas Gerais Energy Company (CEMIG), Brazil. The values were also converted to US$ currency.

Table S1:Paramaters of BPAC, operation of pyrolysis and costs.

BPAC parameters					
BPAC	TA (°C)	IR	PT (min)	$S_{BET-predicted}$	Yield (%)
400/2/60	400	2	60	1037	37
600/1.3/90	600	1.3	90	1411	27

Operational parameters	
**Parameters**	**Value**
Basis of calculus (1 batch) (g)	15
Muffle power (kW)	3.5
Energy Eficiency Rating	0.8
Heating Rate (°C min^{-1})	10
Initial temperature of heating (°C)	25

Costs			
Unit Cost	Low	Intermediate	High
$ZnCl_2$ (US$ g^{-1})	0.011	0,017	0.026
Electric energy (US$ kW^{-1})	0.159	0.163	0.167

The Equations S13 – S19 presents the expressions used for calculate all costs involved with reactant and electricity.

$$THR = \frac{(TA - IT)}{HR} \quad (S13)$$
$$TT = THR + PT \quad (S14)$$
$$RC = BC \times IR \times Unit\ Cost\ ZnCl_2 \quad (S15)$$
$$MC = \frac{Po}{EER} \times \frac{TT}{60} \times Unit\ Cost\ Electric\ Energy \quad (S16)$$
$$TC = RC + MC \quad (S17)$$
$$TA = S_{BET} \times Y \times BC \quad (S18)$$
$$SC = \frac{TC}{TA} \quad (S19)$$

Where THR is the time of heating rate of muffle furnace (min); TA is the temperature of activation (°C); IT is the initial temperature of heating (considered 25 °C); HR is the heating rate of muffle furnace (°C min^{-1}); TT is the total time of muffle furnace operation (min); PT is the pyrolysis time (min); RC is the reactant costs based on $ZnCl_2$ (US$); BC is the basis of calculus, considered as one batch of 15 g of banana Pseudostem; IR is the impregnation ratio; MC is the costs related to muffle furnace (US$); Pot is the power of muffle furnace (kW); EER is the energy efficiency rating (considered as 0.8); TT is the total time of muffle furnace operation (min); TC is the total costs (US$); TA is the total area of a BPAC (m^2); S_{BET} is the specific area of BPAC (m^2 g^{-1}); Y is the yield of BPAC; SC is the specific cost of a BPAC area (US$ m^{-2}).

Author Information

Corresponding Author:

Alisson Carraro Borges. Email: borges@ufv.br.
